# From gluten to therapeutics: molecular mechanisms of celiac disease and emerging alternatives to the gluten-free diet

**DOI:** 10.3389/fphar.2026.1900029

**Published:** 2026-09-14

**Authors:** Federico Manai, Marialaura Amadio

**Affiliations:** 1 Department of Biology and Biotechnology “L. Spallanzani”, University of Pavia, Pavia, Italy; 2 Department of Drug Sciences, University of Pavia, Pavia, Italy

**Keywords:** celiac disease, gene editing, gluten, gluten-free diet, immune response modulation, microbiota, pharmacological strategies, repurposing

## Abstract

Celiac disease (CD) is a chronic immune-mediated disorder triggered by gluten ingestion in genetically predisposed individuals. Increasing knowledge regarding gluten-derived peptides, intestinal barrier dysfunction, innate and adaptive immune responses, and host–microbiota interactions has substantially improved our understanding of disease pathogenesis and stimulated the development of novel therapeutic strategies. The present review aims to provide an integrated overview of the biochemical, immunological, clinical, and therapeutic aspects of CD by linking its molecular mechanisms to the rationale underlying emerging treatment approaches. Particular attention is devoted to the roles of toxic and immunogenic gluten peptides, tissue transglutaminase, intestinal permeability, and immune activation as potential therapeutic targets. Although the gluten-free diet remains the only established treatment, long-term adherence is often challenging and may negatively affect the nutritional status and quality of life while posing an economic burden. Consequently, several alternative or adjunctive approaches have been investigated, including gluten-degrading enzymes, transglutaminase 2 inhibitors, immune-targeted therapies, microbiota modulation, genetically modified cereals, and other experimental interventions. Current evidence suggests that several of these strategies show promising preclinical or early clinical results; however, most of these approaches are limited by insufficient efficacy data, small clinical studies, regulatory challenges, or lack of long-term safety information. At present, none of these approaches can replace the gluten-free diet in routine clinical practice. Overall, the future management of CD will likely require personalized and potentially combined therapeutic approaches. Further translational and clinical studies are needed to identify safe, effective, and accessible alternatives capable of complementing or reducing the burden of lifelong dietary treatment.

## 1 Gluten-related disorders

The consumption of cereals and gluten-containing foods has always played a key role throughout history, and these foods remain some of the main dietary components in Western countries today. As a result, gluten-related disorders (GRDs) have become increasingly relevant, affecting ∼5% of the global population and showing an increasing trend (Elli et al., 2015). Globally, the number of individuals following a gluten-free diet (GFD) for various reasons is also increasing because of the belief that these products are healthier and lower in calories, can help control unclear and diverse symptoms and disorders, or can treat conditions unrelated to the gastrointestinal (GI) system, such as polycystic ovary syndrome (Skoracka et al., 2021) and arthritis (Bruzzese et al., 2021). The term “gluten-related disorders” refers to conditions that arise in certain individuals after ingesting gluten-containing foods and those that do not involve only the GI tract. These conditions can be classified into three main categories based on their origin: immune-mediated, allergic, and non-immune-mediated and non-allergic (Biesiekierski, 2017). Each category can then be subdivided into specific conditions, as shown in Figure 1.

**FIGURE 1:**
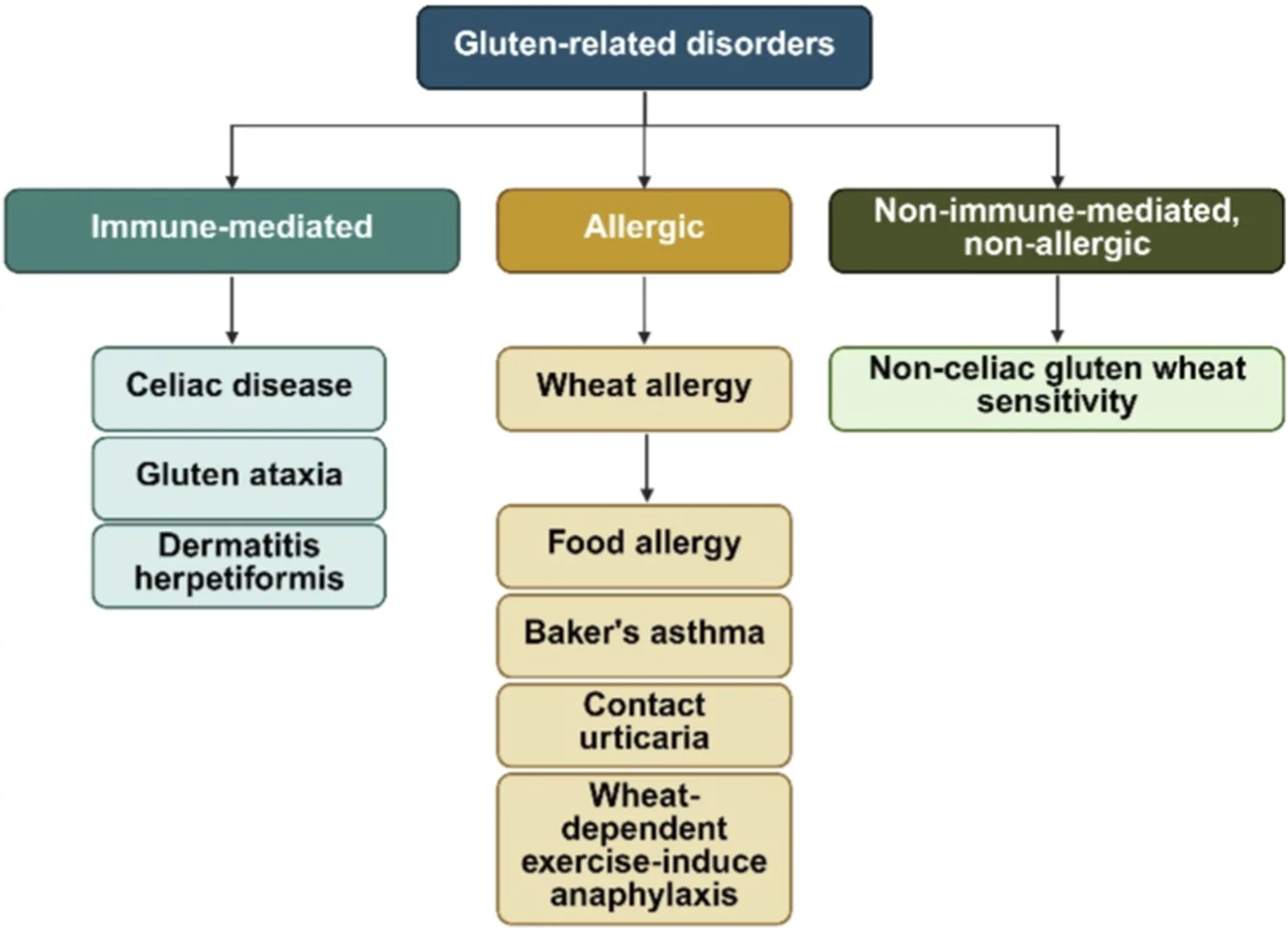
Schematic representation of the conditions referring to the term “gluten-related disorders.” Created in BioRender by author Amadio, M. (2026) (https://BioRender.com/2of9s7g).

### 1.1 Gluten: origin and biochemical properties

The term “gluten” is derived from the Latin word *gluten* (meaning “glue”) and is generally used to refer to a family of hundreds of proteins found in the endosperm cells of developing cereal grains. Since its discovery in 1728 by Jacopo Bartolomeo Beccari, the consumption of gluten has increased significantly in modern diets because it is possible to extract and use it as a food additive to improve the organoleptic qualities. Consequently, gluten is present not only in cereals and bakery products but also processed meats, plant-based meat substitutes, ice cream, candy, condiments, and even medicines (Shewry and Halford, 2002).

Gluten consists of storage proteins linked by interchain disulfide bonds and is rich in proline and glutamine residues (called prolamins) that are present in mature seed endosperms of grains belonging to the *Triticeae* group (i.e., wheat, barley, and rye) and all their hybrids, such as spelt, kamut, and triticale. In wheat, the most extensively studied species is the hexaploid *Triticum aestivum* L. (2n = 42, genome *AABBDD*). These storage proteins accumulate in the grain endosperms during seed maturation and serve as nutrient reserves during germination (Shewry and Halford, 2002; Yan et al., 2014; Bewley, 1997). Based on their botanical origin, prolamins are referred to by different names: gliadins and glutenins from wheat, hordeins from barley, secalins from rye, and avenins from oat (Wieser, 2007). The gliadins and glutenins of wheat show different properties: gliadins are monomeric alcohol-soluble components, whereas glutenins are polymeric alcohol-insoluble components. These proteins can be subdivided into different groups according to their amino acid sequences, compositions, and molecular weights as well as the presence of sulfur. Gliadins can be divided into ω (ω5-, ω1,2-), α/β, and γ subtypes, whereas glutenins are divided into high molecular weight (HMW) (x and y) and low molecular weight (LMW) (B, C, and D) subtypes (Table 1) (Shewry and Halford, 2002; Wieser, 1996).

**TABLE 1:** Gluten protein types. The S-poor and S-rich classifications indicate the sulfur content of the gluten proteins. These data are modified from Shewry and Halford (2002) and Wieser (1996).

Type	Molecular weight (kDa)	Proportion (%)	Classification
ω-Gliadins (ω5, ω1,2)	39–55	3–7	S-poor
α/β-Gliadins	28–35	28–33	S-rich
γ-Gliadin	31–35	23–31	S-rich
x-HMW-glutenin	83–88	4–9	HMW
y-HMW-glutenin	67–74	3–4	HMW
LMW-glutenin (type B)	30–39	19–25	S-poor
LMW-glutenin (type C)	30–39	19–25	S-poor
LMW-glutenin (type D)	30–39	19–25	S-poor

Prolamins share a common evolutionary origin and display similar structural features, including glutamine- and proline-rich repetitive domains that contribute to their biochemical properties (Shewry and Halford, 2002; Napier et al., 1992). In wheat (*T. aestivum* L.), the prolamins are encoded by multigene families located mainly on chromosomes 1 and 6. The gliadins are encoded by the *Gli* loci, whereas the glutenins are encoded by the *Glu* loci. Specifically, the α-gliadins are mainly encoded by the *Gli-2* loci, while the γ- and ω-gliadins are encoded by the *Gli-1* loci. The HMW and LMW glutenins are encoded by the *Glu-1* and *Glu-3* loci, respectively (Shewry et al., 2003). Among the wheat prolamins, gliadins (particularly α-gliadins) are considered the main pathogenic components in susceptible individuals. Their high proline content confers resistance to GI proteases, resulting in the persistence of partially digested peptides that play central roles in the pathogenesis of gluten-related disorders (Biesiekierski, 2017).

#### 1.1.1 Gluten-derived peptides and their pathogenic roles

CD and dermatitis herpetiformis (DH) are caused by the ingestion of gluten and related peptides. Specifically, the pathologic consequences are triggered by the gluten-derived peptides produced during digestion of gliadins and glutenins in the GI tract. These peptides are classified as cytotoxic or immunogenic; specifically, a peptide is considered cytotoxic if it induces epithelial damage in biopsy-derived *in vitro* models or *in vivo*, whereas it is considered immunogenic if it stimulates T cells derived from the intestine or blood of CD patients. Most of the pathogenic peptides are derived from gliadins, which are the toxic components of gluten, while other epitopes may be derived from LMW and HMW glutenins (Table 2) (Shewry et al., 2003). The enzymatic activity of transglutaminase 2 (TG2) plays a key role in the toxicity and immunoreactivity of gluten-derived peptides since these modifications promote binding with HLA-DQ molecules (Molberg et al., 1998; van de Wal et al., 1998). The characteristics of two paradigmatic immunogenic and/or toxic peptides are discussed below.

**TABLE 2:** List of toxic and immunogenic peptides derived from gliadins.

Sequence	Position	Toxicity (0–3)	Immunogenicity (0–3)
VPVPQLQPQNPSQQQPQEQ	α3–21	1	0
VRVPVPQLQPQNPSQQQPQ	α1–29	0	1
QNPSQQQPQEQVPLVQQQ	α11–28	0	1
QVPLVQQQQFPGQQQPFPPQ	α21–40	0	1
PGQQQPFPPQQPY	α31–43	1	0
PGQQQPFPPQQPYPQPQPF	α31–49	1	1
FPGQQQPFPPQQPYPQPQPF	α30–49	0	1
PGQQQPFPPQQPYPQPQPFPSQQPY	α31–55	1	0
QPYPQPQPFPSQQPYLQL	α41–58	0	1
PQPQPFPSQQPY	α44–55	1	0
PQPFPSQQPYLQLQPFPQ	α46–63	0	1
SQQPYLQLQPFPQPQLPY	α51–70	1	0
LQLQPFPQPQLPYPQPQLPY	α56–75	1	0
PQPQLPYPQPQLPY	α62–75*	0	1/3
QLQPFPQ	α57–63*	0	3
QLQPFPQPQLPY	α57–68*	0	1/3
LQLQPFPQPQLPYPQPQLPYPQPQLPYPQPQPF	α57–89*	0	1/3
QLQPFPQPQLPY	α58–69*	0	1/3
PQPQLPYPQPQLPY	α63–76*	0	1/3
PFRPQQPYPQPQPQ	α93–106*	0	1
LIFCMDVVLQ	α123–132	0	1
QQPLQQYPLGQGSFRPSQQNPQAQG	α198–222	0	1
QQYPLGQGSFRPSQQNPQA	α202–220	1	0
QYPLGQGSFRPSQQNPQA	α203–220*	0	1
LGQGSFRPSQQN	α206–217	1	1 (if*)
PSGQGSFQPSQQ	α205–216*	0	1/3
QGSFQPSQQN	α208–217*	0	0/3
LQPQQPFPQQPQQPYPQQPQ	γ60–79	0	1
FPQQPQQPYPQQPQ	γ66–78	0	1
FSQPQQQFPQPQ	γ102–113*	0	0/1
OQPQQSFPEQQ	γ134–153*	0	0/3
VQGQGIIQPQQPAQL	γ222–236*	0	0/1

Toxicity and immunogenicity are ranked from 0 to 3 (0 = none, 1 = low, 2 = medium, and 3 = high); * indicates deamination by TG2. These data are modified from Ciccocioppo et al. (2005).

#### 1.1.2 33-mer

The 33-mer peptide (LQLQPFPQPQLPYPQPQLPYPQPQLPYPQPQPF, residues 57–89), found exclusively in the α2-gliadin coded by the D-genome of *T. aestivum*, is the most immunogenic gliadin peptide (Molberg et al., 2005) that consists of six overlapping copies of three distinct epitopes (Arentz-Hansen et al., 2000; Sollid et al., 2012):

- DQ2.5-glia-α1a (PFPQPQLPY);
- DQ2.5-glia-α2 (PQPQLPYPQ);
- DQ2.5-glia-α1b (PYPQPQLPY).

Compared to the other epitopes, DQ2.5-glia-α2 chiefly drives the 1:1 stoichiometric and stable binding to HLA-DQ2 (Xia et al., 2005; Xia et al., 2006). This interaction depends on the 33-mer and its natural type II polyproline helical conformation. Before binding, TG2 mediates 33-mer deamination, and the resulting deaminated 33-mer preferentially binds HLA-DQ2 at extracellular pH (∼6) (Qiao et al., 2004). At pH 5.5–7.5, the deaminated 33-mer exchanges with the preloaded peptides more efficiently than the monovalent peptides, non-deaminated 33-mer, or tight-binding references, thereby exhibiting a lower dissociation rate that underlies its high HLA-DQ2 affinity (Xia et al., 2005). Conversely, truncated 33-mer-derived peptides can displace soluble/purified HLA-DQ2 with lower EC_50_ than the full peptide, inducing the release of TNF-α in Caco-2 cells via PKA activation (Xia et al., 2006; Laparra Llopis and Sanz Herranz, 2010). *In vitro* and *in vivo* studies confirm that the 33-mer resists gastric, pancreatic, and intestinal proteases (Shan et al., 2002). Similar to the cytotoxic p31-43 peptide, 33-mer mimics lipopolysaccharide action in mouse primary macrophages by inducing MyD88-dependent proinflammatory genes, causing zonulin release, altering the intestinal permeability, and overexpressing TNF-α, IL-12, IP-10, and MCP-5 to recruit monocytes and T cells (Thomas et al., 2006). Additionally, the hydrophobic effects, intramolecular hydrogen bonding, and Gln-generated hydrogen bonds drive concentration-dependent 33-mer self-assembly at pH 7 and lower, reflecting the resistance to the digestive environment. The proline residues maintain the flexible polyproline type II secondary structures crucial for this polymerization, which forms supramolecular structures (>220 nm) that trigger macrophage NF-κB overexpression via the TLR2/TLR4 pathways to promote IP-10, CXCL10, and TNF-α release (Herrera et al., 2014; Herrera et al., 2015; Herrera et al., 2016; Herrera et al., 2018a; Herrera et al., 2018b; Amundarain et al., 2019). According to *ex vivo* studies, 33-mer alters the functions of dendritic cells (DCs) in CD patients and controls treated with different peptides (i.e., 8-mer, 19-mer, and 33-mer), thus increasing gut-homing CCR9^+^ expression on autologous T cells (Escudero-Hernández et al., 2020). In animal models, dietary gluten is correlated with increased Th17 cells in the pancreatic lymph node, cytokine homeostatic loss, and altered DC populations in type 1 diabetes (T1D; the most common CD comorbidity) (Antvorskov et al., 2012; Antvorskov et al., 2013; Adlercreutz et al., 2014; Antvorskov et al., 2014; Larsen et al., 2015). This impact on T1D onset appears to be mediated by the 33-mer; when administered orally in T1D mice, these fragments exploit the prediabetic intestinal permeability to reach the exocrine pancreas, where they decrease KATP-channel activity to stimulate resting and glucose-stimulated insulin secretion (Bruun et al., 2016; Dall et al., 2013). Similar immunogenic fragments exist in other proteins like γ-gliadin, notable among which is the GI-enzyme-resistant 26-mer (γ5-gliadin; FLQPQQPFPQQPQQPYPQQPQQPFPQ) containing three epitopes (DQ-γ-III, DQ-γ-VI, and DQ-γ-VII). Overall, computational proteome analyses have helped identify 60 putative peptides sharing 33-mer and 26-mer characteristics (Shan et al., 2005). The role of the 33-mer as the major immunodominant gluten peptide is strongly supported by biochemical, immunological, and clinical evidence. Conversely, several proposed mechanisms, including peptide self-assembly, TLR activation, and extraintestinal effects, are mainly supported by *in vitro* or animal studies and require further validation in humans.

#### 1.1.3 p31-43

The p31-43 peptide (PGQQQPFPPQQPY) contained in the 25-mer peptide (p31-55) is the best-characterized cytotoxic peptide derived from α2-gliadin. Similar to the 33-mer, p31-43 spontaneously aggregates in aqueous solutions at 3 < pH < 8, exhibiting polyproline II and β-sheet-like conformations (Herrera et al., 2020). These properties confer resistance to both GI enzymes and intracellular degradative processes, resulting in disrupted autophagy homeostasis (Manai et al., 2019; Manai et al., 2018; Comincini et al., 2017). In the absence of specific receptors, p31-43 is internalized via endocytic vesicles; however, its sequence homology with the hepatocyte growth-regulator substrate (HRS) protein, a key component of endocytic trafficking, allows it to interfere with vesicle maturation (Barone and Zimmer, 2016; Paolella et al., 2018; Barone et al., 2010). This endocytic pathway impairment triggers multiple significant consequences. It causes transferrin receptors to accumulate on the cell membrane alongside elevated IL-15 levels. Furthermore, *in vitro* and *in vivo* evidence show that p31-43 induces PCK-mediated cytoskeletal rearrangements via the zonulin signaling pathway to increase intestinal permeability. Concurrently, these cytoskeletal modifications are driven by the inhibition of EGFR inactivation and promote enterocyte proliferation, leading to crypt hyperplasia and villous atrophy (Barone et al., 2010; Clemente et al., 2003; Barone et al., 2007). Beyond EGFR, the binding of α-gliadin peptides to a glutamate receptor, a heat shock protein, and the chemokine receptor CXCR3 increases intestinal permeability, with CXCR3 and MyD88 activation being specifically involved in enhanced zonulin release (Lammers et al., 2008). Gliadin peptides additionally induce oxidative stress to provoke inflammatory responses and DNA damage. The accumulation of p31-43 in lysosomes elevates intracellular levels of reactive oxygen species (ROS), which activate TG2. This enzyme promotes various enzymatic and non-enzymatic intracellular reactions linked to physiological and pathological events. For instance, p31-43-mediated ROS accumulation triggers the ubiquitination and proteasomal degradation of PPAR-γ, evoking an inflammatory response (Agnihotri and Mehta, 2017; Luciani et al., 2010). This TG2-mediated PPAR-γ downregulation may also be enhanced by p31-43-induced direct endoplasmic reticulum and mitochondrial stresses, which trigger Ca^2+^ release (Caputo et al., 2012). Furthermore, p31-43-driven ROS accumulation accounts for the increased DNA damage observed *in vitro* and *in vivo* when using whole digested gliadins (Monguzzi et al., 2019). Gliadin peptides trigger inflammation in various cell types through two additional mechanisms, namely, the MyD88/TRIF/NF-kB and NLPR3/ASC/Caspase-1 activation pathways, where both lead to the release of proinflammatory cytokines, i.e., mature IL-1β (Palova-Jelinkova et al., 2013; Gómez Castro et al., 2019). Immunologically, p31-43 drives an innate rather than adaptive immune response because it cannot bind HLA-DQ2 molecules or T cell receptors (TCRs) (Calvanese et al., 2019). *In vitro* and *ex vivo* data demonstrate that p31-43 induces epithelial HLA-DQ2 expression and elevates IL-15, CD83, COX-2, and CD25 levels in the *lamina propria* cells (Maiuri et al., 1996; Maiuri et al., 2003). The underlying vesicle trafficking delay contributes to the trans-presentation of increased IL-15 on the cell membrane via IL-15Rα, while also playing a role in the interferon (IFN)-α-mediated induction of TLR7 and subsequent MyD88/MAPK/NF-κB activation. Finally, IL-15 release acts synergistically with epidermal growth factor to promote enterocyte proliferation of the crypts (Barone et al., 2011; Nanayakkara et al., 2018; Lania et al., 2019; Nanayakkara et al., 2013), while p31-43 concurrently activates macrophages and mast cells that are both critical to CD pathogenesis (Thomas et al., 2006; Frossi et al., 2017). Unlike the 33-mer, p31-43 primarily appears to contribute to innate immune activation and epithelial stress responses. However, the relative importances of the numerous mechanisms proposed, including impaired endosomal trafficking, oxidative stress, autophagy defects, and inflammasome activation, remain unclear as most evidence has been derived from experimental models.

#### 1.1.4 Gluten exorphins

In addition to immunogenic and cytotoxic peptides, other gluten-derived peptides may contribute to CD pathogenesis. Gluten exorphins (GEs) are small peptides generated by the GI digestion of gluten and casein that act as agonists of opioid receptors. Their morphine-like activity was first demonstrated by Zioudrou et al. (1979) through naloxone-mediated inhibition of adenylate cyclase (AC), inhibition of mouse *vas deferens* contraction, and displacement of opioid ligands from rat brain receptors. Subsequent studies in dogs showed a naloxone-reversible increase in postprandial insulin levels following administration of digested gliadins, while oral administration of digested gluten in humans increased intestinal transit and somatostatin-like immunoreactivity, both of which were also reversed by naloxone (Schusdziarra et al., 1981; Morley et al., 1983). Some researchers subsequently isolated and characterized several GEs, including A4, A5, B4, B5, and C5, using enzymatic digestion of gluten and opioid bioassays (Fukudome and Yoshikawa, 1992; Fukudome and Yoshikawa, 1993; Fukudome et al., 1997); A4 and A5 preferentially bind to the δ-opioid receptor (DOR), whereas B4 and B5 interact with both δ- and μ-opioid receptors, with B5 representing the most potent peptide. These peptides are resistant to GI proteolysis and may be released by pancreatic elastase during digestion. Administration of purified A5 and B5 increases postprandial insulin secretion, whereas intracerebroventricular administration of B5 elevates prolactin levels. Another peptide, gliadorphin-7, induces naloxone-sensitive neuronal activation in specific brain regions (Fukudome et al., 1995; Fanciulli et al., 2002; Fanciulli et al., 2004; Sun and Cade, 2003). DOR is a seven-transmembrane receptor encoded by the *ORPD* gene and activates multiple intracellular signaling pathways, including AC, phospholipases, ERK1/2, AKT, c-Jun, and Ca^2+^ signaling. Importantly, DOR expression increases during inflammatory processes, including intestinal inflammation (Gendron et al., 2016; DiCello et al., 2018; Cahill et al., 2003; Pol et al., 2003; Pol and Puig, 2004).

On this basis, GEs have been proposed as possible contributors to asymptomatic CD, while the opioid-mediated effects on immune cells have resulted in hypotheses regarding their involvement in enteropathy-associated T-cell lymphoma (EATL), small bowel carcinoma, and neuropsychiatric manifestations, including anxiety, depression, and schizophrenia (Pruimboom and de Punder, 2015; Plein and Rittner, 2018; Eisenstein, 2019; Debruyne et al., 2010; Szczepaniak et al., 2020; Shahabi et al., 2006; Manai et al., 2023a; Eaton et al., 2004; Pynnönen et al., 2005; Bressan and Kramer, 2016). However, although GEs exhibit clear opioid activities in experimental models, their contributions to CD pathogenesis remain highly speculative. Most available evidence has been derived from biochemical studies, animal experiments, or indirect physiological observations, whereas direct evidence in patients with CD is limited. Likewise, the proposed associations with asymptomatic CD, neuropsychiatric disorders, and intestinal malignancies remain largely hypothetical. Therefore, GEs should currently be regarded as an emerging area of research rather than an established pathogenic mechanism.

### 1.2 Wheat allergy

Wheat allergy (WA) is an adverse immune reaction that is either immunoglobulin E (IgE)- or non-IgE-mediated and directed against wheat proteins. Its prevalence in Europe is estimated to be approximately 0.1% based on food challenge studies, although higher rates have been reported in self-reported surveys (Nwaru et al., 2014). The clinical manifestations of WA may include anaphylaxis, urticaria, angioedema, acute asthma, rhinitis, enteritis, and dermatitis. Similar to other food allergies, IgE-mediated WA is characterized by a shift from regulatory T cells (Tregs) toward a Th2 response driven by DC expression of OX40L and amplified by basophils and type 2 innate lymphoid cells. IL-4 promotes IgE class switching and suppresses Treg activity, whereas the clinical symptoms result from IgE-mediated activation of mast cells and basophils through the FcεRI receptors (Tordesillas et al., 2017). In contrast, the mechanisms underlying non-IgE-mediated WA remain incompletely understood and may involve lymphocytic or eosinophilic inflammation leading to intestinal or cutaneous manifestations, such as atopic dermatitis, eosinophilic enteritis, and eosinophilic esophagitis (Cianferoni and Spergel, 2009).

Several clinical forms of WA have been described, including baker’s asthma and wheat-dependent exercise-induced anaphylaxis (WDEIA). Baker’s asthma and rhinitis are occupational diseases caused by inhalation of wheat allergens, including α-amylase inhibitors, agglutinins, peroxidases, and lipid transfer proteins (LTPs) (Tatham and Shewry, 2008). WDEIA is a rare but potentially life-threatening condition characterized by anaphylaxis triggered by wheat ingestion and subsequent physical exercise. Multiple IgE-binding epitopes have been identified in gliadins, glutenins, and LTPs, with ω5-gliadin representing the major allergen involved in WDEIA (Matsuo et al., 2004; Battais et al., 2005; Denery-Papini et al., 2011; Matsuo et al., 2005; Matsuo et al., 2015). Differences in epitope recognition among the clinical phenotypes suggest that both protein conformation and route of exposure influence the immune responses to wheat allergens (Laurière et al., 2007). Diagnosis of WA remains challenging: specific IgE measurements and skin prick tests are commonly used but have limited predictive value, making oral food challenge the diagnostic gold standard despite the risk of severe reactions. More recently, molecular allergy diagnostics and basophil activation tests have emerged as promising *in vitro* tools to improve the diagnostic accuracy and reduce the need for provocation tests (Elli et al., 2015; Kleine-Tebbe and Jappe, 2017; Santos et al., 2021).

### 1.3 Non-celiac gluten sensitivity

Non-celiac gluten sensitivity (NCGS) is a syndrome characterized by both intestinal and extra-intestinal symptoms triggered by the ingestion of gluten-containing foods in individuals who are not affected by CD or WA (Catassi et al., 2015). Its clinical presentation frequently overlaps with irritable bowel syndrome (IBS) and occurs in the absence of IgE, CD-specific antibodies, or HLA-DQ2/DQ8 positivity. The global prevalence of NCGS remains uncertain because many individuals often initiate a GFD without medical supervision. The common symptoms include abdominal pain, bloating, altered bowel habits, fatigue, headache, joint pain, and skin manifestations. Although the pathogenesis remains poorly understood, the symptoms have been attributed to gluten, amylase-trypsin inhibitors (ATIs), and fermentable oligosaccharides, disaccharides, monosaccharides, and polyols (FODMAPs), whose relative contributions remain controversial. Intestinal biopsies from NCGS patients show increased expression of Toll-like receptors (TLR1 and TLR2) and reduced expression of the Treg marker FOXP3, supporting the involvement of innate immunity (Sapone et al., 2011).

FODMAPs are osmotically active short-chain carbohydrates, including fructose, lactose, fructans, galactans, sorbitol, and mannitol (Shepherd et al., 2013). Several studies suggest that they may account for the symptoms in a subset of patients, raising questions regarding the specific role of gluten in NCGS. Preliminary evidence also shows microbiota alterations, characterized by reduced *Bacteroidetes* and increased *Firmicutes*. Although low-FODMAP diets appear to have limited effects on the overall microbiota composition, reductions in *Bifidobacteriaceae* have been observed; probiotic supplementation has also shown the potential to partially restore these alterations, although its clinical significance remains uncertain (Skodje et al., 2018; Biesiekierski et al., 2013; Staudacher et al., 2017; Dieterich et al., 2019). ATIs have been proposed as potential contributors to symptom generation. These plant defense proteins comprise several subtypes, including chloroform-methanol extractable proteins, which act as potent TLR4 activators. *In vitro* studies demonstrate that ATIs stimulate monocytes, macrophages, and DCs to release proinflammatory mediators, whereas murine studies show increased duodenal inflammation through TRL4–MD2–CD14 signaling pathways involving MyD88 and TRIF (Hellinger and Gruber, 2019; Junker et al., 2012; Schuppan and Zevallos, 2015). However, most mechanistic evidence has been derived from experimental models, and their relevance in human disease remains uncertain.

NCGS remains difficult to diagnose due to the lack of validated biomarkers, and its diagnosis is currently based on the exclusion of CD and WA. Although first described in the 1980s, its true prevalence remains unknown, and it appears to occur primarily in young and middle-aged women (Cárdenas-Torres et al., 2021). The management of NCGS generally involves a physician-supervised GFD or low-FODMAP diet, but these treatment recommendations are not standardized and the need for lifelong gluten avoidance remains debated. Furthermore, some authors have suggested that the nocebo effects and widespread perception of gluten as harmful may contribute to symptom development in a subset of patients (Dale et al., 2019). Overall, NCGS should be regarded as a heterogeneous clinical entity with incompletely understood pathophysiology. The respective contributions of gluten, FODMAPs, ATIs, microbiota alterations, and nocebo effects remain unclear, while the lack of standardized diagnostic criteria continues to limit both clinical practice and epidemiological estimates.

### 1.4 Dermatitis herpetiformis (DH)

DH is a disorder comprising a specific cutaneous manifestation of CD. The predisposing factors of DH are the same as those of CD. The major and characteristic symptoms are the presence of itching, subepidermal vesicles, and blistering rash as well as accumulation of neutrophils. The estimated prevalence of DH is about 0.01% (Bolotin and Petronic-Rosic, 2011a). The small intestine of DH patients are characterized by IgA deposits against TG2; however, the skin lesions contain inflammatory infiltrates characterized by granular deposits of IgA against epidermal TG3. This cross-reactivity could be due to the high avidity of IgA antibodies that had originally increased against TG2 in the small bowel (Salmi et al., 2014; Sárdy et al., 2002; Bolotin and Petronic-Rosic, 2011b). The immune mechanism that mediates the onset of DH is still not understood, but it is clear that the clinical skin manifestation is sustained by the action of neutrophils. These cells show increased expression of CD11b and FcIgA receptors as well as decreased expression of L-selectin. The migration of neutrophils to the accumulation sites of IgA complexes (probably mediated by IL-8) and the binding of their Fc receptor to immunoglobulin lead to activation of these immune cells (Smith et al., 2002; Hall et al., 2006). DH is diagnosed by analysis of the serum anti-TG2 levels and direct immunofluorescence of skin lesions against IgA anti-TG3 deposits. The only effective therapies for DH are adherence to a GFD and administration of topical drugs, such as dapsone, to reduce the skin rash (Bolotin and Petronic-Rosic, 2011b).

### 1.5 CD

CD is defined as a chronic systemic immune-mediated disorder that primarily affects the small intestines of genetically predisposed individuals upon triggering by the ingestion of gluten and by other possible environmental factors (Di Sabatino and Corazza, 2009). Currently, about 1% of the global population is affected by this condition, but several studies show that it is actually widely underdiagnosed (Caio et al., 2019). The clinical manifestations can vary greatly and make it difficult to correctly diagnose CD. The most common symptoms are steatorrhea, weight loss, malabsorption of micronutrients, and malnutrition; however, there are other symptoms that do not exclusively affect the GI tract, which are referred to as extra-intestinal symptoms. The diagnosis of CD is based on a combination of the patient’s clinical history, blood tests, and duodenal biopsies through gastroduodenoscopy. The only effective treatment available currently is a strict GFD, which must be followed rigorously to prevent long-term complications, such as nutritional deficiencies, diabetes, thyroiditis, infertility, osteoporosis, and increased risk of cancer (specifically EATL and small intestine carcinoma), leading to a general increase in mortality (Elli et al., 2015).

#### 1.5.1 Epidemiology and comorbidities of CD

Between 0.5% and 2% of the global population is diagnosed with CD. Populations in certain countries represent exceptions: higher percentages are detected in Sweden (3%) and among the Saharawi people (5%–6%), whereas percentages lower than 0.5% are reported in East Asia (e.g., Japan, Vietnam, and Indonesia). The low prevalence of CD in East Asia is critically linked to the limited consumption of gluten-containing cereals and a low genetic predisposition, particularly a low frequency of human leukocyte antigen (HLA) class II haplotypes (specifically *HLA-DQ2*), which are mainly involved in the development of the disease. However, experts argue that these estimates may be heavily confounded by underdiagnosis due to atypical or asymptomatic clinical presentations in these regions (Lionetti et al., 2015).

The prevalence of CD in the Saharawi population, namely, those living in the Western Sahara region between Morocco, Algeria, and Mauritania, is the highest in the world. This is attributable to a genetic predisposition along with the fact that this population has been forced into a sudden change in eating habits since the 1970s, when it was displaced to southern Algeria owing to the invasion by Morocco and owing to the humanitarian aid that introduced gluten to people who were not accustomed to consuming it (Catassi et al., 1999). It has been observed that the prevalence of this disease may vary even within the same country. For example, in India, the prevalence of CD is lower in the southern regions (0.1%) compared to the northern regions, especially in the Punjab area (1.2%) located at the northern border along Pakistan. The reasons for this variability are still unknown: since the prevalence of the *HLA-DQ2* and *HLA-DQ8* haplotypes is similar throughout the country, the disease prevalence is hypothesized to be correlated with the average daily intake of wheat, which is higher in the north (Ramakrishna et al., 2016). Moreover, there is limited data regarding the disease prevalence in developing countries, but it is likely that the prevalence is increasing even in these areas because of the spread of Western dietary habits (Lionetti et al., 2015).

CD is more common in females than males, with a ratio of 1.85:1. While the global prevalence of CD is traditionally estimated to be between 0.5% and 2%, systematic reviews and meta-analyses underscore a significant rising trend in global incidence, especially over the last 50 years, driven by not only environmental shifts but also the widespread availability of high-sensitivity serological screening. In contrast to the historical view of CD as a predominantly Western disease, contemporary epidemiological models demonstrate that the prevalence in developing countries is escalating rapidly. This phenomenon is strictly correlated with the global transition toward Westernized dietary habits and industrialized food production. However, a major limitation in current epidemiological literature is the lack of standardized registry data in developing regions, which has led to a severe underestimation of the true global burden, often referred to as the “celiac iceberg” (Catassi et al., 2022).

Initially, CD was considered as a typical childhood disease because it is more common in children than adults, when considering the age of the population. However, this claim was disproved by Vilppula et al. (2008), who argued that this is likely a coincidence due to the probability that older individuals remain seronegative compared to younger ones. Moreover, detection of CD in the elderly population may be due to loss of gluten tolerance rather than a delayed diagnosis. CD is a multifactorial disorder caused by interactions among the genetic, epigenetic, and environmental factors, which can influence the incidence and spread (Makharia et al., 2022).

Several studies have shown that the risk of death can increase from 1.2 to 2 times, even among individuals adhering to a GFD (Ludvigsson, 2012). Therefore, despite better knowledge and the wider availability of gluten-free products, the affected patients still face a small but significant increase in risk of death compared to the general population. The higher mortality seems to be mainly due to the risk of developing non-Hodgkin lymphoma. However, it has been shown that the long-term risk of mortality is significantly lower for individuals diagnosed with the disease during childhood. In agreement with this observation, a recent systematic review with meta-analysis on the cause-specific and all-cause mortality in CD compared to the general population based on studies between 1970 and 2022 revealed that the mortality in CD is increased predominantly due to cancers, particularly non-Hodgkin lymphoma, although differing significantly across disease phenotypes. However, the mortality of patients with CD has decreased significantly in recent decades, likely because of improvements in disease diagnosis and management (Maimaris et al., 2024).

CD is often associated with other autoimmune diseases: type 1 diabetes, IgA deficiency, Hashimoto’s thyroiditis, Addison’s disease, connective tissue diseases (particularly Sjogren’s syndrome), chromosomal diseases (e.g., Down, Turner, and Williams syndromes), neurological diseases (e.g., peripheral neuropathy, epilepsy, cerebellar ataxia), DH, liver diseases (e.g., primary biliary cholangitis, hepatitis, and primary sclerosing cholangitis), and idiopathic dilated cardiomyopathy. Additionally, other systemic disorders with various extra-intestinal manifestations are common (Sollid and Thorsby, 1993), as shown in Figure 2.

**FIGURE 2:**
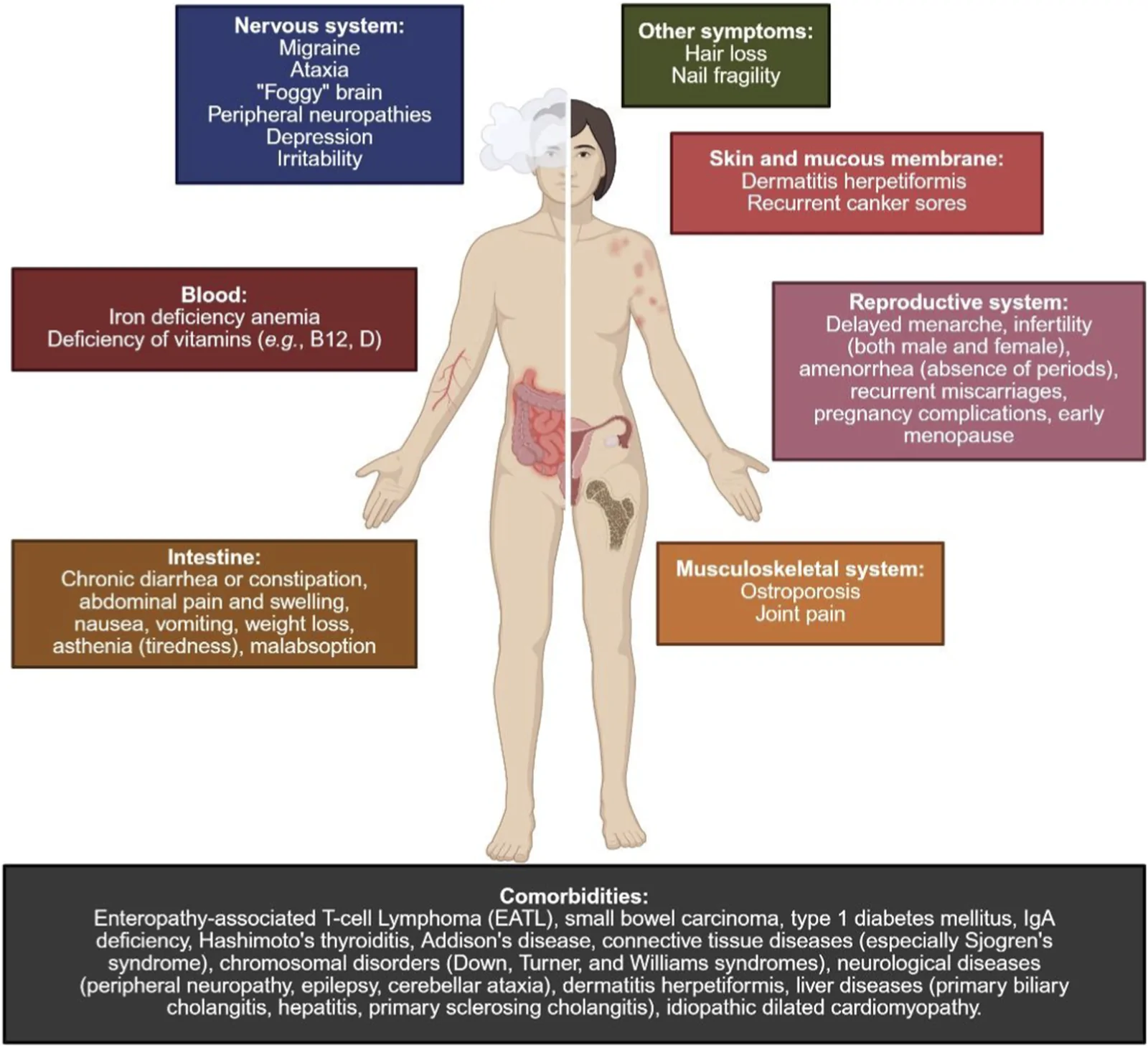
Schematic representation of celiac disease (CD) symptoms and comorbidities. Created in BioRender by author Amadio, M. (2026) (https://BioRender.com/6lgis0j).

### 1.5.2 Genetics

CD is characterized by a non-Mendelian pattern of inheritance involving HLA and non-HLA genes. The *HLA-DQ* alleles localized on chromosome 6 (6p21.3), as determined by genetic linkage analysis, are the primary genetic predisposing factor for CD. This gene encodes the α and β chains of the major histocompatibility complex (MHC) class II, which is involved in antigen presentation from antigen-presenting cells (APCs) to CD4^+^ T cells. Most of the patients have the DQ2 variant (DQA1*05-DQB1*02), while a minority group has the DQ8 variant (DQA1*03-DQB1*0302) (Sollid and Thorsby, 1993; Sollid et al., 1989). Since the *HLA-DQ* locus is carried by approximately one-third of the Caucasians, other non-HLA genes with roles in CD pathogenesis have been investigated. Thus, several factors have been identified, as shown in Table 3.

**TABLE 3:** Non-HLA genes associated with CD.

Localization	Gene	Reference
5q31-q33	Cytokine gene cluster	Greco et al. (1998)
2q33.2	*CTLA4*	Hunt et al. (2005)
19p13.1	*MYO9B*	Van Belzen et al. (2003)
4q27	*KIAA1109, TENR, IL2, IL21*	van Heel et al. (2007)
1q31	*RGS1*	Hunt et al. (2008)
2q11-q12	*IL18RAP, IL18R1*	Hunt et al. (2008)
3p21	*CCR1, CCR2, CCL2, CCR3, CCR5, XCR1*	Hunt et al. (2008)
3q25-q26	*IL12A*	Hunt et al. (2008)
3q28	*LPP*	Almeida et al. (2014), Huang et al. (2017)
6q25.3	*TAGAP*	Hunt et al. (2008), Huang et al. (2017)
12q24	*SH2B3*	Hunt et al. (2008)

All the identified non-HLA genes play roles in immunity, inflammation, and cytoskeletal modifications. A second-generation genome-wide association study helped identify other genes implicated in immune functions that could influence the onset and pathogenesis of CD: *BACH2*, *CCR4*, *CD80*, *CIITA-SOCS1-CLEC16A*, *ICOSLG*, *ZMIZ1*, *EST1*, *RUNX3*, *THEMIS*, and *TNFRSF14* (Dubois et al., 2010). Although numerous non-HLA loci are associated with CD susceptibility, their individual effect sizes are generally modest. In contrast, HLA-DQ2/DQ8 remains the major genetic determinant of disease risk. Most non-HLA variants converge on pathways involved in immune regulation rather than disease-specific mechanisms, and their clinical relevance remains largely uncertain.

### 1.5.3 Pathophysiology

Gluten ingestion and genetic predisposition play key roles in the development of CD, but they are not the only factors involved. Almost all patients express HLA class II, specifically the *HLA-DQ2*/*HLA-DQ8* haplotypes or both, which are also expressed in about 40% of the healthy populations in America, Europe, and Asia. This means that these allelic variants are necessary but not sufficient for the development of CD. Therefore, other factors should be considered even though their effects and mechanisms are not yet fully understood. Some studies have analyzed the diets of children, particularly the roles of breastfeeding and introduction of gluten-containing foods during weaning, but given the negative results, the European Society for Paediatric Gastroenterology Hepatology and Nutrition (ESPGHAN) still recommends introducing gluten between 4 and 12 months in small amounts initially. In contrast, viral infections of the digestive system, such as those caused by adenovirus, hepatitis C virus (Plot and Amital, 2009), or reoviruses (Bouziat et al., 2017), seem to be risk factors. Bacterial infections, such as those caused by *Campylobacter* (Riddle et al., 2013), can also pose a risk for this condition. Furthermore, the use of certain medications, such as antibiotics (e.g., penicillins, macrolides, and quinolones) (Mårild et al., 2013; Guarner et al., 2024) and proton pump inhibitors (Lebwohl et al., 2014; McMillan et al., 2023), is implicated in the onset of CD, likely owing to their modifications in the gut microbiota and environment.

In agreement, healthy intestinal microbiota represent a protective factor that decreases the risk of developing the disease, as first shown *in vivo* in a study conducted on germ-free (GF) mice, specific-pathogen-free (SPF) mice colonized with a microbiota free of opportunistic pathogens and proteobacteria, and conventional SPF mice. However, the limitation of this model is that there are no naturally celiac mice, so transgenic mice must be used, as described in the study (Galipeau et al., 2015). Studies have also been conducted to evaluate the roles of microbiomes/microbiota in humans, and such studies have increased in the last few years (Catassi et al., 2024; Stanciu et al., 2024). Since the intestinal microbiota is particularly sensitive to environmental factors, it has been observed that its composition differs in each person, especially among untreated celiac patients, those following a GFD, and non-celiac individuals. For example, it has been demonstrated that the microbiotas of untreated celiac patients have higher fecal concentrations of *Bifidobacterium bifidum* than healthy individuals and that there is a higher incidence of Gram-negative bacteria in celiac children (Lebwohl et al., 2018). Moreover, it was found that opportunistic pathogens and duodenal commensal bacteria have different abilities to metabolize gluten, thus affecting its pathogenicity. Specifically, *Pseudomonas aeruginosa* is one of the opportunistic pathogens that generates peptides that can cross the intestinal barrier more easily (Caminero et al., 2016).

Other researchers later demonstrated through duodenal biopsies that untreated celiac patients have greater proteolytic activities against gluten peptides than healthy control individuals. This was correlated with the abundance of proteobacteria, including *P. aeruginosa*. These authors also showed that *P. aeruginosa*, which expresses elastase that degrades gluten (elastase B, LasB), can induce more severe inflammation in mice expressing genetic risk factors for CD or even be associated with moderate villous atrophy. It is therefore likely that proteases expressed by opportunistic pathogens influence the host’s immune responses (Caminero et al., 2019). Together, these observations suggest that environmental factors may modulate disease risk in genetically susceptible individuals. However, the strength of evidence differs substantially among the proposed triggers. Viral infections and microbiota alterations currently show the strongest mechanistic support, whereas the contributions of antibiotics, proton pump inhibitors, and early feeding practices remain less clearly established.

The pathophysiological mechanisms involved are summarized in Figure 3 and begin with the ingestion of gluten that is only digested partially. Specifically, among the various undigested gliadin peptides, two are characterized best: p31-43 (contained within the longer 25-mer peptide and predominantly cytotoxic) and the 33-mer (immunodominant peptide) (Frossi et al., 2019). These peptides can cross the epithelial barrier of the small intestine more easily and reach the *lamina propria mucosae* because the tight junctions between enterocytes lose their integrity in CD due to upregulation of zonulin—a human protein capable of modulating the junctions and consequently intestinal permeability (Fasano, 2012). Additionally, undigested peptides can penetrate the intestinal barrier through transcytosis or retrotranscytosis of secretory IgA via the transferrin receptor CD71 (Di Sabatino and Corazza, 2009). Once the peptides reach the *lamina propria mucosae*, tissue transglutaminase (tTG2) located on the brush border epithelium or in the extracellular space of the subepithelial region catalyzes a deamination reaction to convert glutamine residues into glutamate, thus conferring a negative charge on the peptides. This reaction is a key event in the pathogenesis of CD as it increases the immunogenicity of gliadins and triggers subsequent reactions. The deamidated peptides, particularly the 33-mer, are then recognized by HLA-DQ2 or HLA-DQ8 molecules on the APCs containing positively charged amino acid residues. This activates both the adaptive and innate immune responses in celiac individuals.

**FIGURE 3:**
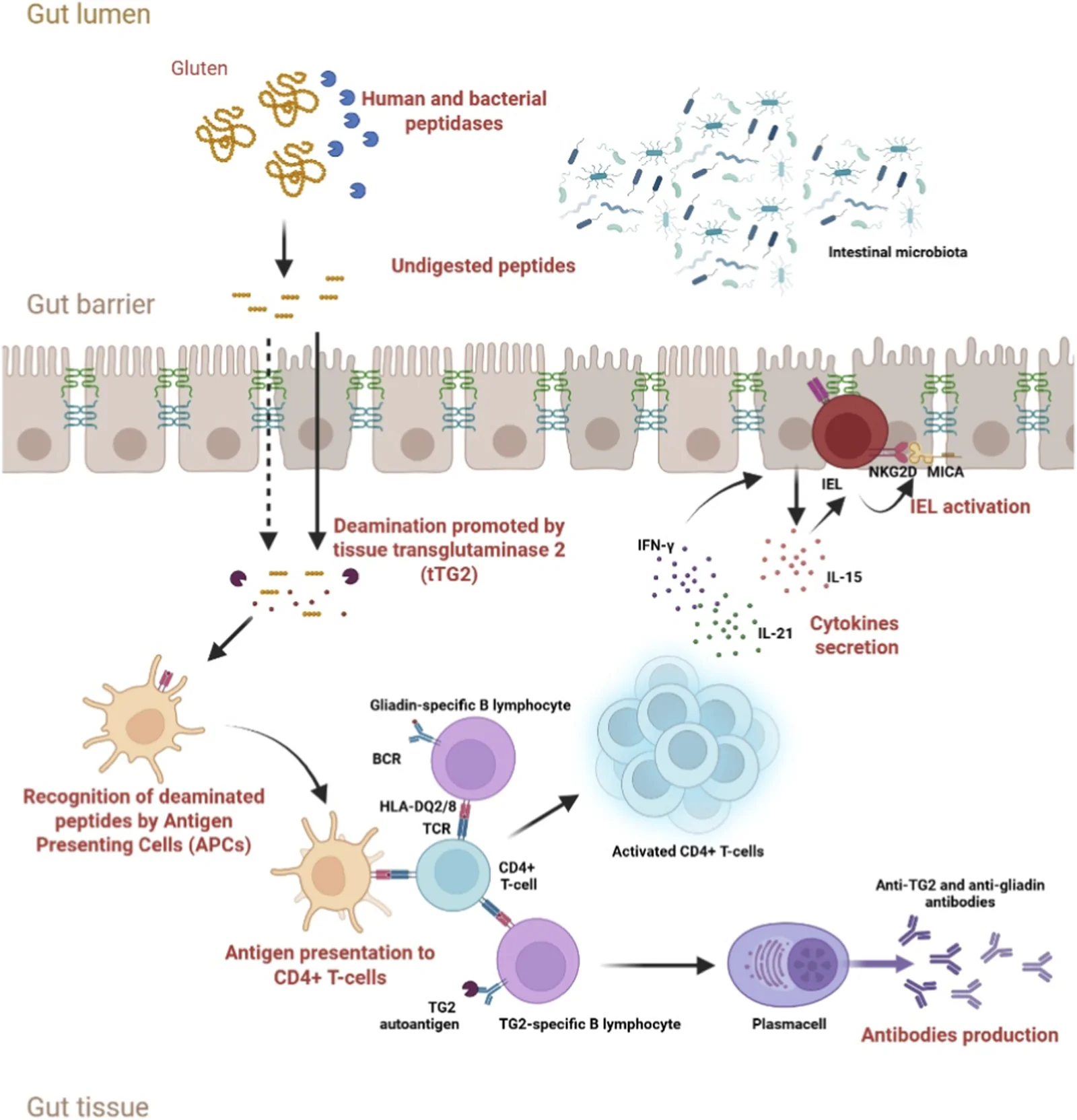
Representation of the pathophysiology of CD. Created in BioRender by author Amadio M. (2026) (https://BioRender.com/n6exjhg).

The undigested peptides are presented by the HLA molecules on APCs to CD4^+^ T cells in the *lamina propria*. These T cells are then activated and start producing high levels of proinflammatory cytokines, particularly IFN-γ. In turn, the proinflammatory cytokines activate helper T cells (Th1) to promote increased cytotoxicity of the intraepithelial lymphocytes (IELs) and natural killer (NK) cells, which cause apoptosis of the enterocytes through the Fas/Fas ligand (FasL) system. Additionally, the Th1 cells favor the release of IL-15, which induces strong cytotoxic activity, especially from the NKG2D cells. NKG2D is an activating receptor belonging to the NKG2 family of lectin-like receptors, whose main ligands are the MHC class I chain-related proteins A and B (MICA and MICB) that serve as autoantigens induced by stress (Hüe et al., 2004).

The proinflammatory cytokines produced by CD4^+^ T cells also simultaneously activate the helper T cells (Th2), which activate and promote differentiation of B lymphocytes into plasma cells secreting anti-gliadin and anti-tTG2 antibodies, whose measured levels are used to diagnose the disease (Sharma et al., 2020). Current evidence strongly supports TG2-mediated deamidation, HLA-DQ2/DQ8 presentation, and adaptive T cell activation as the central events in CD pathogenesis. In contrast, the contributions of microbiota alterations, innate immune pathways, peptide aggregation, and environmental modifiers remain incompletely understood. Hence, future studies are needed to clarify the interactions of these mechanisms and whether they represent causal factors or secondary phenomena.

### 1.5.4 CD classification and symptoms

The clinical spectrum of CD exhibits profound interindividual variability, which led to the establishment of the Oslo consensus nomenclature in 2011. Accordingly, CD is divided into different categories as classic, non-classic, asymptomatic, potential, subclinical, and refractory (Ludvigsson et al., 2013). However, the application of these criteria in contemporary clinical practice remains a subject of debate. While the consensus discouraged confusing terms like “typical” or “atypical,” recent literature emphasizes a significant shift in the disease’s clinical paradigm, as reported by Di Sabatino and Corazza (2013). In their view, each statement in the Oslo classification should have undergone a more thorough evaluation to ensure greater precision and persuasiveness:

- Classic CD: It is more common in children under the age of three and is characterized by chronic diarrhea, loss of appetite, abdominal distension, and growth delay. Bloating, constipation, and abdominal pain are more typical in older children and adults. Malabsorption is quite rare, while symptoms resembling IBS are more frequent.
- Non-classic CD: This form affects both children over the age of three and adults. It is characterized by non-specific symptoms, such as recurrent abdominal pain, recurrent aphthae stomatitis, dental enamel defects, constipation, osteopenia or osteoporosis caused by altered absorption of vitamin D and calcium, and iron-deficiency anemia (found in 40% of cases). Changes in reproductive functions, such as late menarche, amenorrhea, recurrent miscarriages, premature birth, early menopause, and changes in sperm count and mobility, are often linked to this form of CD.
- Silent/asymptomatic CD: The intestinal mucosa is damaged in this type of CD, but there is no clear symptomatology. This form is often identified only through serological screening in individuals at risk, namely, those who have first-degree relatives with CD or those affected by other autoimmune diseases.
- Refractory CD (RCD): This form affects about 0.3%–0.4% of patients. In these individuals, the symptoms and villous atrophy persist even after 12 months of strict GFD, thus posing higher risks to general health. There is no valid treatment; however, type I refractory CD (RCD I) responds well to corticosteroid therapy (such as budesonide) and immunosuppressive drugs (such as infliximab), while type II RCD or RCD II (characterized by the presence of aberrant T cells in the small intestine expressing NK receptors and IL-15) does not respond well to such treatments (Kivelä et al., 2021).

However, these criteria do not fill the gap of the clinical paradox and shift in CD phenotype: indeed, contemporary epidemiological data reveal that the “classic” manifestation has transitioned into a minority presentation in developed countries. Instead, the clinical landscape is now overwhelmingly dominated by the “non-classic” and “asymptomatic” forms, which frequently manifest in adulthood and thus represent a major diagnostic hurdle.

### 1.5.5 Diagnosis

The increase in the number of CD diagnoses has been made possible by advances in research and the development of new technologies and diagnostic tools. The first significant contribution was the introduction of intestinal biopsy in 1955, while serological tests for measuring specific antibodies were introduced around 1980. Prior to this, diagnosis was based solely on clinical symptoms (Ghazanfar et al., 2023). Since the development of CD is characterized by the presence of serum autoantibodies, measurement of anti-tTG2 antibodies in combination with or preceded by the measurement of total serum IgA (to exclude selective IgA deficiency) is considered the best first-line test at present. Various methods are used to determine the presence of autoantibodies (Table 4): enzyme-linked immunosorbent assay, immunofluorescence, radio immuno assay, and chemiluminescence. The test for measuring anti-endomysial antibodies (EMAs) has a specificity of close to 100%; however, it is not considered as a valid first-line screening approach owing to the difficulty in standardizing the methodology, as the interpretation of results can vary from one operator to another. The measurement of antibodies against deamidated gliadin peptides (DGPs) is among the other diagnostic tests available, although it is less commonly used.

**TABLE 4:** Serological tests used to diagnose CD. The data are compiled from Rubio-Tapia et al. (2013) and Kelly et al. (2015).

Test	Mean sensitivity	Mean specificity	Use
Anti-tTG IgA	94%	97%	Test
Anti-tTG IgG	Variable (12%–99%)	Variable (86%–99%)	IgA deficiency
Anti-EMA IgA	>90%	99%	Confirmatory test
Anti-DGP IgG	>90%	>90%	IgA deficiency and children <2 years
HLA-DQ2/DQ8	91%	54%	Confirmation

If the anti-tTG2 antibodies are positive along with the EMA test, there is a high probability of CD. A gastroduodenoscopy is then performed to collect at least two biopsy samples from the duodenal bulb and four samples from the descending portion of the duodenum; these tissues are analyzed to confirm or exclude the diagnosis by considering various degrees of compromise, ranging from lymphocytic infiltration to complete villous atrophy. The hallmarks present in CD patient biopsies are IEL infiltration, villous atrophy, and crypt hyperplasia. Currently, a cutoff value of 20–25 IELs/100 enterocytes is considered as suggestive of CD. Villous atrophy is the consequence of intestinal inflammation; the histopathological changes originally categorized by Marsh (1992) and Oberhuber et al. (1999) were simplified by Catassi et al. (2022) and Corazza et al. (2007) as follows:

Marsh score modified:

- A: non-atrophic
- B1: atrophic, villous–crypt ratio < 3:1
- B2: atrophic, villi no longer detectable

While histological confirmation via duodenal biopsy (under normal diet) was historically considered the undisputed gold standard, current diagnostic algorithms are the subject of intense international debate. The 2020 ESPGHAN guidelines established a robust framework for a non-biopsy approach in pediatric populations with anti-tTG2 titers ≥10 times the normal upper limit along with a positive EMA test. Although this strategy reduces pediatric endoscopic risks and costs by half, its translation to adult clinical practice remains highly controversial. Historically, major international societies, including the American College of Gastroenterology (ACG), have strictly mandated confirmatory biopsies in adults owing to the poor standardization of commercial serological kits and necessity to exclude mimicking enteropathies. However, contemporary literature highlights a shifting paradigm that was partly driven by endoscopic constraints during the COVID-19 pandemic, where several adult guidelines evaluated non-biopsy protocols for highly selected adult cohorts. This ongoing debate emphasizes that while a non-biopsy strategy is highly specific under strict criteria, universal consensus on its safety in adults has not yet been reached (Rubio-Tapia et al., 2013; Husby et al., 2020).

Given the significant burden of the “celiac iceberg,” the clinical community is actively debating whether to replace the current “case-finding” strategy with population-wide screening programs. However, mass screening poses complex health-economics and clinical dilemmas. Critics argue that the public health resources would be inefficiently allocated as a single population-wide serological screening fails to account for the dynamic nature of CD, which can develop at any age. Consequently, a longitudinal screening approach would be required to capture late-onset cases, thus increasing healthcare costs exponentially. Therefore, contemporary consensus continues to favor a low-threshold case-finding strategy targeted at high-risk groups provided that clinician awareness across non-gastroenterological specialties is significantly enhanced to mitigate diagnostic delays.

## 2 GFD

Currently, a strict and lifelong adherence to a GFD remains the cornerstone of management for CD. Clinically, adherence to a GFD is highly effective in driving symptom resolution and reducing the risk of long-term comorbidities. However, contemporary evidence hints that the therapeutic efficacy of a GFD is far more nuanced and limited than previously assumed, particularly in adult-onset patients. In 95% of children, a strict GFD can restore normal intestinal conditions within 2 years, while in adults, 34% recover within 2 years and 66% recover within 5 years (Aljada et al., 2021). The persistent villous atrophy despite strict dietary adherence outlines the major clinical challenge of non-responsive celiac disease (NRCD). To ensure patient safety, international regulations, such as the Codex Alimentarius (in document CDX 118-1979) and EU Regulation 828/2014, have standardized the threshold for “gluten-free” labeling at <20 mg/kg (ppm). Although clinical trials confirm that a daily intake below 10 mg of gluten is safe for the majority of individuals, the persistent debate surrounding controversial ingredients like oats highlights the difficulty of achieving universal dietary standardization (Kivelä et al., 2021).

## 2.1 GFD-related issues

Although the GFD is effective and resolves most cases of CD, it has been shown to be complex and challenging to follow for a lifetime. Additionally, there remain many uncertainties and questions for which there are no definitive answers, such as safe levels of gluten exposure. Studies have shown that the exposure levels have high inter-individual variability, but it has been concluded that a daily intake of less than 10 mg is unlikely to cause significant histological abnormalities (Crepaldi et al., 2023). Since gluten is a widely used ingredient, having to follow a diet that requires its complete elimination involves a significant change in eating habits. Ensuring the absence of gluten, following a balanced diet, and reducing social restrictions and burdens for patients are the main challenges that have emerged. A study by Shah et al. (2014) showed that participants with CD perceive the GFD treatment as more impactful, both economically and health-wise, compared to participants with other chronic conditions that may actually be more debilitating, such as kidney failure, heart failure, IBS, or gastroesophageal reflux disease. Moreover, evaluating the GFD requires a balanced consideration of its significant metabolic and nutritional limitations. From a nutritional standpoint, it is preferable to emphasize naturally gluten-free foods—such as meat, fish, fruits, and vegetables—which are rich in essential micronutrients. In contrast, a GFD largely based on processed products presents several drawbacks, including vitamin D, iron, calcium, folate, and fiber deficiencies, along with excess intake of low-quality fats and sugars. These imbalances can have important health consequences: inadequate consumption of fiber, folate, and zinc is associated with increased risks of cardiovascular disease and cancer, whereas excessive intake of sugars and fats is linked to a higher risk of diabetes, cardiovascular disease, and obesity (Kreutz et al., 2020).

One of the major factors affecting adherence to a GFD is its social burden, which can also affect the psychological well-being of patients and their families. In our daily lives, food holds significant value as it is a fundamental element of sharing and socializing with others. Because of the dietary restrictions on celiac individuals, this becomes a real limitation, leading to changes in social life and causing isolation, regardless of whether these were chosen or imposed. Several studies report that the fear of not finding suitable food, risk of contamination, or even avoiding the feeling of being different can cause people with CD to prefer abstaining from visiting restaurants and traveling; this may lead to the development of anxiety, eating disorders, isolation, and therefore a general compromise in the quality of life (Aljada et al., 2021). Another issue related to the GFD is the cost as the entire production and distribution chain of gluten-free products is more complex, making these foods more expensive than those containing gluten.

In this regards, it is advisable to schedule regular check-ups after the diagnosis, typically every 12 months, to monitor progress and wellbeing, evaluate patients’ adherence to the GFD, and provide adequate support by involving not only the doctor but also a dietitian. The necessity of performing follow-up intestinal biopsies remains debated: most experts consider it reasonable to perform them 1–2 years after starting treatment, particularly for patients over 40 years old or those still exhibiting symptoms (Catassi et al., 2022). Ultimately, the complex balance between clinical benefits and severe socio-nutritional limitations highlights that a GFD is an incomplete solution, underscoring the critical necessity for non-dietary supportive therapies currently under clinical evaluation.

## 3 Transgenic gluten-free plants and regulatory limitations

The use of gluten-free cereals in food production represents a possible alternative to the GFD. Over the last decade, several research groups have attempted to develop a wheat variety with a reduced percentage of immunogenic peptides using traditional mutagenesis and plant selection. However, none of these groups succeeded in developing a low-immunogenic wheat variety that is safe for CD patients (Verma et al., 2021). Moreover, advancements in genetic engineering have enabled the application of RNA interference (RNAi) technology to reduce the expressions of α-gliadins, γ-gliadins, and ω-gliadins in wheat. Among all the wheat lines produced with this method, the E82 line showed the best results, with a 98% reduction in gluten content (Gil-Humanes et al., 2010). Furthermore, bread made from the flour of this line was tolerated by subjects with NCGS (Haro et al., 2018) and did not trigger any immune responses in a double-blind randomized study involving celiac patients (Guzmán-López et al., 2021).

Using the CRISPR/Cas9 method, researchers at the Institute for Sustainable Agriculture in Córdoba managed to deactivate up to 35 of the 45 total genes responsible for producing gliadins, thereby reducing their toxicity by 85% (Sánchez-León et al., 2018). Both the RNAi method and CRISPR/Cas9 system are highly effective in creating wheat lines without the toxic epitopes for celiac patients, but the latter offers greater advantages: it allows deactivation of unwanted genes, resulting in stable and inheritable mutations without the need to express a transgene, meaning that the phenotype is independent of environmental conditions. Additionally, it could be used to eliminate larger chromosomal fragments containing multiple genes and replace highly immunogenic fragments with less toxic ones (Marín-Sanz et al., 2026; Marín-Sanz et al., 2025; Yu et al., 2024). However, this method also presents obstacles like the RNAi approach, particularly regulatory limitations due to the requirement for genetic modification and transgenic expression.

Under Directive 2001/18/EC, the Court of Justice of the European Union has established that plants produced by genetic editing are regulated as genetically modified organisms (GMOs). In contrast, other countries have decided not to regulate genetically edited plants as GMOs if there is no foreign DNA in the final product. As a result, genetically modified wheat lines containing hypoimmunogenic gluten and products derived from them can be developed and marketed, but these are not accepted within the European Union unless GMO safety tests have been conducted, which can cost more than 100 million euros. Therefore, this decision has negative and significant impacts on the application of this technique, companies producing such products, and patients themselves (Jouanin et al., 2020).

Despite these promising advances, several translational challenges remain. Long-term safety in patients with CD has not yet been established, and even substantial reductions in the immunogenic peptides may not completely eliminate the risk of immune activation. Furthermore, modifications of gluten proteins may affect the technological properties of wheat and consequently its industrial applicability. Finally, regulatory restrictions, high development costs, and limited public acceptance of genetically modified crops continue to represent important barriers to clinical and commercial implementations. Notably, to date, no hypoimmunogenic wheat variety has been approved for consumption by patients with CD.

## 4 Hookworm infestation

Potential non-pharmacological strategies include experimental infection with parasitic helminths, which are multicellular organisms (specifically worms) that improve immune responses by altering the intestinal permeability and providing potential benefits for certain autoimmune diseases, such as CD (Croese et al., 2015). Among parasitic helminths, those belonging to the *Ancylostomatidae* family have been used, specifically hookworms such as *Ancylostoma duodenale* and *Necator americanus* that colonize the human intestine exclusively (Kucik et al., 2004). However, only the latter has been exploited to test its immunomodulatory activity in individuals with CD.

Daveson et al. (2011) analyzed the effects of *N. americanus* infestation in a 21-week double-blind placebo-controlled study involving 20 adults with well-controlled CD on a GFD and naïve (never exposed) to helminths; this study was conducted at the Princess Alexandra Hospital in Brisbane (protocol number NCT00671138 in ClinicalTrials.gov; status: completed). Gradual skin inoculations were performed with 10 or 5 hookworm larvae or placebo, respectively, at week 0 and week 12. At week 20, wheat equivalent to 16 g of gluten per day was administered orally for 5 d. Colonization with hookworms was confirmed in all 10 cases, with transiently painful enteritis occurring in 5 of them. Chronic infestation was asymptomatic, and the mucosa infested by hookworms maintained a healthy appearance. The study revealed that infestation by *N. americanus* was overall safe, although some participants exhibited symptoms. Furthermore, it allowed the testing of effects at various levels, including the Marsh duodenal score and quantification of systemic IFN-γ-producing cells. In both groups, the wheat challenge exacerbated these factors, demonstrating that helminth infestation did not offer any benefits against CD.

Croese et al. (2015) conducted a 52-week study involving 12 adult celiac patients adhering to the GFD. Here, 20 larvae of *N. americanus* were inoculated, followed by administration of increasing doses of gluten consumed as pasta: from 10 to 50 mg for 12 weeks (microchallenge), 25 mg per day + 1 g twice a week for 12 weeks (GC-1g), and 3 g per day for 2 weeks (GC-3g). Each participant developed parasitic infestation, which was confirmed by detecting eggs in the stool. Two subjects withdrew from the study after the microchallenge because they no longer met the necessary criteria; ten participants completed the GC-1g test, and eight of these participants also completed GC-3g. The results showed that the median ratio of villus height to crypt depth did not decrease after GC-1g as expected, while the mean titers of anti-tTG2 antibodies decreased, contrary to the expected increase after GC-3g. An improvement in the quality-of-life score was observed, while the symptom indexes, intraepithelial lymphocyte percentages, and Marsh scores remained unchanged. T cells in the intestine expressing IFN-γ were reduced, with corresponding increases in the CD4+-Foxp3+ regulatory T cells. The study concluded that infestation by *N. americanus* stabilized, if not improved, all gluten toxicity indexes tested in celiac patients, thus improving gluten tolerance.

Another randomized placebo-controlled study was conducted between March 2017 and October 2019 across three Australian clinics and one New Zealand clinic after obtaining authorization and registering the study (protocol number NCT02754609 in ClinicalTrials.gov; status: completed). Unlike the previous studies, which involved very small numbers of participants, this study included 54 celiac patients and comparison with a placebo. The study lasted 94 weeks and involved treatment with 20 or 40 *N. americanus* third-stage larvae or placebo, followed by increasing gluten consumption (50 mg per day for 12 weeks, 1 g intermittently twice a week for 12 weeks, 2 g per day for 6 weeks, followed by a GFD for 1 year). At week 42, the successful completion rates were similar in each group, and gluten-related adverse events were significantly lower in participants treated with hookworms. However, the villus height and crypt depth worsened compared to baseline values in both groups of infested patients. Additionally, a retrospective analysis revealed that 9 of the 40 participants treated with larvae did not develop the infestation. Despite similar completion rates at week 42 between participants who were positive and negative for hookworms, the quality-of-life scores were lower in the hookworm-positive participants after the intermittent gluten test. In conclusion, hookworm infestation could not restore tolerance to moderate and sustained gluten consumption (2 g/d) but was associated with improved symptom scores after intermittent consumption of lower doses of gluten (Croese et al., 2020).

Overall, the available studies provide conflicting results. While some trials reported modest improvements in symptoms or immunological parameters, others failed to demonstrate protection against gluten-induced mucosal damage. Furthermore, these studies involved small patient populations and heterogeneous protocols. Consequently, hookworm therapy should be considered an experimental immunomodulatory approach rather than a clinically applicable treatment for CD (Alhassan et al., 2019).

## 5 Microbial therapy

Several studies have demonstrated that the intestinal microbiota plays a role in the onset of CD. Microbial therapies, including probiotics and prebiotics, have been proposed in recent years as a potential non-pharmacological strategy for treating the condition. Probiotics are live microorganisms capable of exerting beneficial functions on the body after ingestion (Morelli and Capurso, 2012), whereas prebiotics are indigestible food substances that selectively support the growth and activity of beneficial bacteria already present in the intestinal tract (Pineiro et al., 2008).

The specific mechanisms by which microorganisms protect against the immune responses triggered by gluten vary, including antigen metabolism, enhancement of the intestinal barrier, and modulation of both adaptive and innate immune responses (Caminero et al., 2019). In fact, it has been observed in animal models that certain bacterial strains present in the human GI tract have the ability to degrade gluten, effectively reducing its immunogenicity, while other strains can restore intestinal permeability and suppress adverse responses to food components via Tregs that induce oral tolerance to food antigens (Hiippala et al., 2018). An example of such a strain is the bacterium *Lactococcus lactis*, which has been genetically modified to secrete a deamidated gliadin epitope; after oral administration of *L. lactis* in a transgenic non-obese diabetic AB° DQ8 mouse, induction of antigen-specific tolerance was shown (Huibregtse et al., 2009). However, the efficacy and safety of these bacterial strains need further evaluations in clinical studies.

At the same time, microbial therapies should not be considered as standalone treatments but as adjunct to diet since a GFD alters the homeostasis of the intestinal microbiota, potentially leading to dysbiosis. For commercially available products containing probiotics and prebiotics, specific and sufficient trials have not been conducted to justify their exclusive use in the treatment of CD; however, only studies confirming their safety and validity for addressing common dysbiosis are available. Studies on certain probiotic supplements have been conducted in humans to analyze their potential beneficial effects in CD. In a double-blind, randomized, placebo-controlled study involving 33 pediatric patients newly diagnosed with CD and following a GFD, supplementation with *Bifidobacterium longum* CECT 7347 resulted in a reduction in immune markers, including CD3^+^ T lymphocytes, HLA-DR^+^ T lymphocytes, and certain cytokines such as TNF-α and IL-10, alongside a decrease in the number of *Bacteroides fragilis* bacteria in the stool that typically cause opportunistic infections (Olivares et al., 2014). Another study investigated a 3-month administration of a supplement containing the strains *Bifidobacterium breve* B632 and BR03 in 40 children with CD; this study showed an increase in *Actinobacteria* and a restoration of the ratio between *Firmicutes* and *Bacteroidetes* in the intestinal microbiota. Previous scientific works have highlighted an important imbalance in these bacteria, which is implicated in the pathogenesis of CD, due to reductions in the numbers of *Actinobacteria* and *Firmicutes* following an increase in *Bacteroidetes* (Quagliariello et al., 2016).

Harnett et al. (2016) conducted a multicenter study to test VSL#3 as a powerful multistrain probiotic containing eight strains of lactic acid bacteria and bifidobacteria with the highest CFU per serving available; the test involved 45 adults with CD who had been adhering to a GFD for at least 12 months but reported only partial symptom improvement. However, no differences were observed in symptom severity or the fecal microbiota. In contrast, a double-blind, randomized, placebo-controlled study conducted in 109 patients with CD, who exhibited symptoms similar to those of IBS (which also shares some pathophysiological mechanisms with CD), demonstrated that the administration of a product containing a mixture of five strains of lactic acid bacteria and bifidobacteria for 6 weeks improved the GI symptoms and increased *Staphylococcus* and *Bifidobacterium* levels in the stool. These bacteria are among the strains most affected by the onset of CD and have the greatest ability to restore intestinal permeability (Francavilla et al., 2019).

Prebiotics can stimulate the growth and activity of potentially beneficial bacterial strains for the intestine, particularly *Bifidobacterium* and *Lactobacillus*. However, most of these studies involve prebiotic formulations combined with other ingredients, such that the beneficial effects attributable to prebiotics are not clear. Krupa-Kozak et al. (2017) conducted a randomized placebo-controlled clinical study in pediatric celiac patients adhering to a GFD to evaluate the influence of inulin (a carbohydrate found in various plant foods that is indigestible by human enzymes) enriched with oligofructose and labeled “Orafti®-Synergy1.” They found an increase in *Bifidobacterium* and a decrease in *Lactobacillus* levels in the children treated with inulin compared to those on the placebo, which showed reductions in *Bifidobacterium* species diversity and count.

In conclusion, *Bifidobacteria*, *Lactobacilli*, and substances that promote their development may have the potential to restore intestinal microbiota composition and permeability, while reducing the production of cytokines, production of antibodies, and inflammation associated with gluten intake (Marasco et al., 2020). However, available evidence has been derived from relatively small and heterogeneous studies, which have not demonstrated consistent benefits on mucosal healing or disease control. Therefore, microbial therapies should currently be considered adjunctive and experimental approaches rather than established treatments for CD.

## 6 Pharmacological strategies

As the pathogenic processes of CD are increasingly being understood, various targets have been identified for potential pharmacological therapies for treatment, such as replacement strategies and/or support for the GFD. Based on this knowledge, multiple experimental therapies are being developed, which have allowed several pharmacological candidates to enter clinical trials of phases I, II, and III that have either been completed or are currently ongoing. The main strategies are summarized in Figure 4 and further explained in the following paragraphs.

**FIGURE 4:**
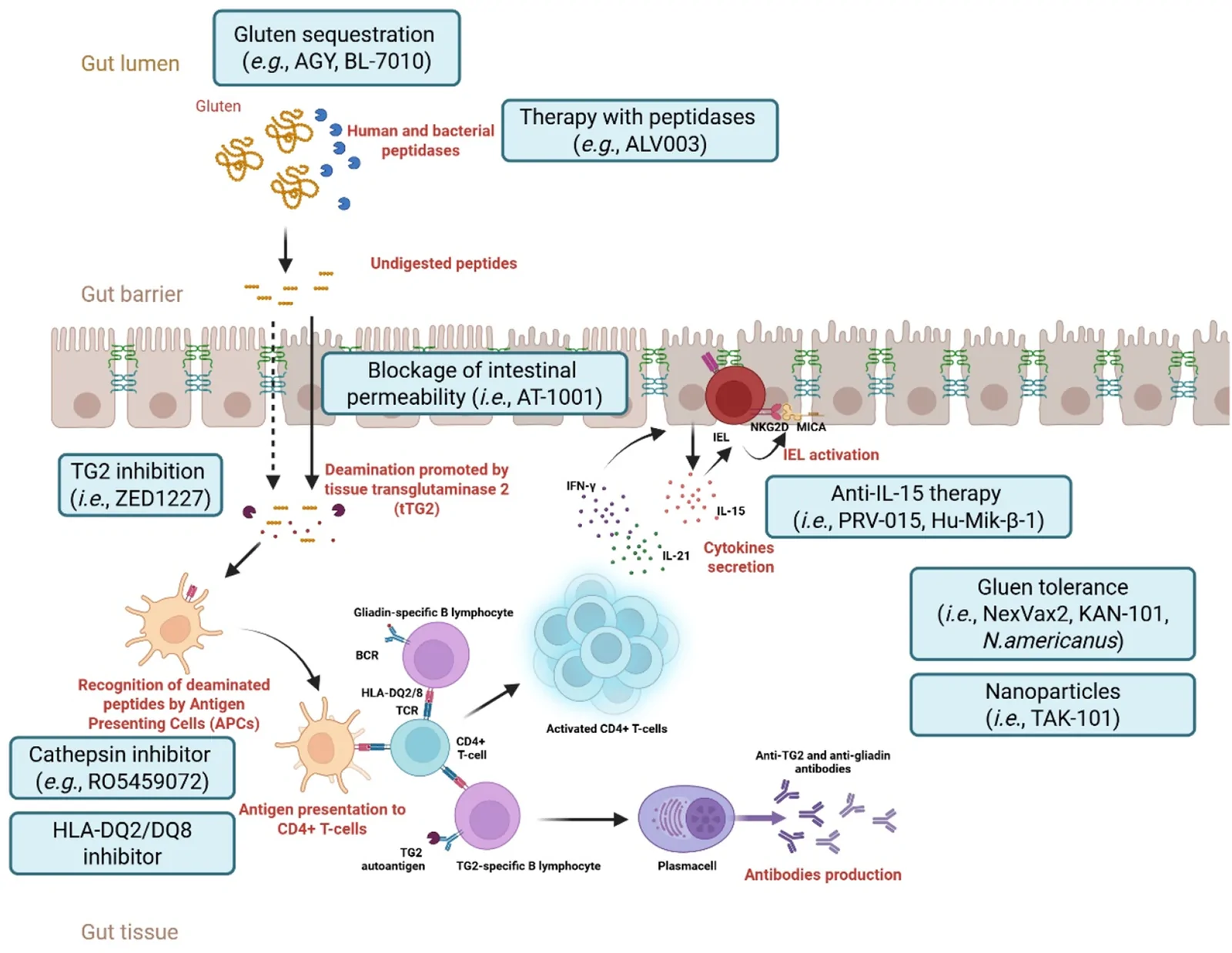
Overview of the pharmacological strategies hypothesized for CD. Created in BioRender by author Amadio M. (2026) (https://BioRender.com/cm2kydm).

### 6.1 Gluten sequestration in the intestinal lumen

The first approach for treating CD involves sequestration of gluten in the intestinal lumen before it undergoes partial digestion into immunogenic peptides. This treatment can be established with various compounds, including polymeric binders and gliadin-targeting antibodies. It has been shown that gliadin-reactive chicken yolk antibodies (AGY) neutralize gliadin and reduce its absorption in a mouse model. In a 6-week, open-label, single-arm phase I study conducted on 10 adults with well-controlled CD on a GFD, oral administration of AGY was found to be safe and potentially associated with disease improvement (Sample et al., 2017). A phase II, crossover, randomized, double-blind, placebo-controlled study lasting 14 weeks and involving 149 patients with CD on a GFD is listed under the ClinicalTrials.gov identifier NCT03707730 (status: completed); the primary outcome of this study was to evaluate the effects of AGY on CD symptoms reported by patients; the secondary objectives were to assess the product’s safety and ability to act on autoantibodies and intestinal permeability. However, the study was completed more than 1 year ago and the results are yet to be published.

Another molecule that is potentially useful in CD is BL-7010—a non-absorbable high-molecular-weight polymer composed of hydroxyethylmethacrylate and sodium 4-styrenesulfonate that binds to α-gliadin. *In vitro* and preclinical studies in gluten-sensitive mouse models have demonstrated specific binding of BL-7010 to gliadins and reduction of its harmful effects (McCarville et al., 2014). A phase I, randomized, double-blind, crossover, placebo-controlled study to evaluate the safety of BL-7010 and its absorption in the bodies of patients with well-controlled CD on a GFD has also been completed; however, the results of this study are not available despite the study being listed as completed in 2014 on ClinicalTrials.gov under the number NCT01990885.

Studies have also shown that polyphenols can similarly sequester gluten proteins and interfere with their enzymatic hydrolysis (Pinier et al., 2012). Polyphenols are organic compounds composed of multiple phenolic groups and represent a structurally distinct class of secondary metabolites produced by plants; they are present in many plant-based foods, and more than 8,000 compounds have been identified so far, including flavonoids, phenolic acids, lignans, and stilbenes (Pandey and Rizvi, 2009). They have been shown to have anti-inflammatory activities in chronic GI diseases and anti-nutritional properties with respect to proteins, limiting their absorption with positive consequences for gluten proteins (Pietta, 2000). These compounds may therefore have beneficial impacts on human health, demonstrating not only anti-inflammatory properties but also antitumor and antiobesity effects both *in vitro* and *in vivo*. Specifically, their effectiveness in protecting against intestinal disorders with etiologies similar to CD has been demonstrated due to their abilities to interact with immune-stimulating proteins, mediate inflammatory responses, regulate the intestinal barrier, and modulate the gut microbiota (Bernardi et al., 2020; Van Buiten and Elias, 2021; Calabriso et al., 2022). In 2017, Van Buiten and Elias provided the first evidence of the biological efficacy of polyphenols in the treatment of CD through a study that characterized gliadin-polyphenol complexes. This study showed that the polyphenols remain stable throughout digestion and prevent gliadin-mediated inflammation and permeability *in vitro*. This study still remains the basis for ongoing research to expand the knowledge and discover various potential mechanisms through which polyphenols can exert protective effects against the symptoms and pathogenesis of CD, while confirming the potential benefits of supplementation for individuals with CD (Van Buiten and Elias, 2021).

Despite encouraging preclinical results, the clinical efficacies of gluten-sequestering compounds remain uncertain. Their effectiveness may depend on the amount of gluten exposure, the timing of administration, and individual variability in intestinal conditions. Furthermore, the long-term safety and ability to prevent mucosal damage under real-life dietary conditions remain to be established. Consequently, these approaches should currently be regarded as adjunctive rather than replacement therapies for the GFD.

### 6.2 Peptidase therapy

Oral enzyme therapy is a widely studied approach that aims to inactivate the immunogenic gluten peptides in the human GI tract through the integration of peptidases, thereby minimizing the peptides that reach the small intestine. However, before their use in clinical practice, peptidases must meet certain requirements: they must be stable in the harsh conditions of the upper GI tract, particularly in the very acidic pH of the stomach; they must effectively degrade the immunogenic gluten peptides in the stomach; they must not have any harmful effects on the patients. The most studied enzymes that meet these requisites and are able to specifically degrade proline-rich gliadin peptides both *in vitro* and *in vivo* belong to the family of prolyl endopeptidases (PEPs) derived from bacteria and fungi, including *Aspergillus niger*, *Flavobacterium meningosepticum*, *Myxococcus xanthus*, and *Sphingomonas capsulata* (Bethune and Khosla, 2012). In particular, the PEPs derived from *A. niger* (AN-PEPs) are able to degrade gluten at acidic pH and are resistant to digestion by pepsin, in addition to being well-tolerated, as demonstrated in a randomized, double-blind, placebo-controlled pilot study involving a total of 16 celiac patients consuming gluten (Tack et al., 2013). Another randomized, double-blind, crossover, placebo-controlled study with 12 healthy volunteers has been reported; here, each volunteer received either a high amount of AN-PEP enzyme or placebo along with a high- or low-calorie meal containing more than 4 g of gluten every 7 d for 4 weeks. The meal was directly infused into the stomach in the subject through a nasoduodenal catheter, from which gastric and duodenal samples were collected at pre-established time points. The concentration of α-gliadin was measured in the samples to verify the degradative efficacy of AN-PEP. It was found that 60 min after administering the meal with the enzyme, there were no traces of gluten in the stomach, in contrast to the placebo group where up to 180 min was required to observe the same effect (Salden et al., 2015). In a subsequent crossover, randomized, placebo-controlled trial conducted by König et al. (2017), 18 subjects with NCGS who consumed 0.5 g of gluten together with AN-PEP or placebo tablets showed that the enzyme significantly degraded most of the gluten in the stomach before it entered the duodenum. However, it remains to be determined whether this enzyme prevents damage to the duodenal mucosa.

In 2017, the EFSA issued a positive opinion on the use of the AN-PEP enzyme Tolerase® G. Currently, an over-the-counter supplement called Galactol® is available, where each gastro-resistant tablet contains 100 mg of Tolerase® G with a combination of α- and β-galactosidase. The recommended dosage is 1–2 tablets before main meals, and the leaflet states that the product “is useful for individuals who have gluten sensitivity, with or without intestinal discomfort due to the ingestion of gluten and/or complex carbohydrates” (EFSA Panel on Dietetic Products, Nutrition and Allergies et al., 2017). As a supplement, this tablet should not be regarded as the sole therapy and has not been approved for use after a celiac diagnosis.

Another group of microbial enzymes known as STAN1 used commonly in dietary supplements and known to have a modest ability to detoxify gluten was tested in a phase II clinical study registered in ClinicalTrials.gov with the identifier number NCT00962182 in 2018. This randomized, double-blind, placebo-controlled trial evaluated the effects of STAN1 in 38 celiac patients whose serology was still positive despite not consuming gluten for at least 6 months. The aim of the trial was to assess the decrease in anti-tTG2 antibody levels after administering a cocktail of enzymes and 500 mg of gluten for 12 weeks. However, the final results are not yet available.

The most studied enzyme mixture in humans is ALV003 or latiglutenase. It is an orally administered mixture of two proteases that degrade gluten and are active in the acidic environment of the stomach: ALV001, a cysteine endoprotease glutamine-specific enzyme derived from germinating barley seeds; ALV002, a modified recombinant version of a PEP from *S. capsulata* (Gass et al., 2007). After a phase I clinical study demonstrated the drug’s tolerance and stability (Siegel et al., 2012), a phase IIa clinical trial was conducted in adult celiac subjects: 20 patients received ALV003 and 21 patients received placebo, with all patients being subjected to daily gluten tests subsequently. Initially, it appeared that ALV003 was able to attenuate the damage caused by a daily dose of up to 2 g of gluten to the small intestine mucosa in patients; however, the GI symptoms, headaches, and fatigue induced by the gluten test did not differ between the treatment groups (Lähdeaho et al., 2014). A phase IIb, double-blind, placebo-controlled study evaluated the efficacy and safety of ALV003 at variable doses in a large cohort of 494 patients, all of whom adhered to a GFD for at least 1 year. The subjects reported moderate or severe symptoms related to possible accidental gluten exposure (e.g., contamination) in the month before trial commencement, and the histological scores or symptoms did not improve in the treated group compared to placebo (Murray et al., 2017). However, the authors of the study reassessed the trial data in a *post hoc* analysis and discovered that celiac patients with positive serology actually showed symptom improvement when taking ALV003 during meals (Syage et al., 2017). In light of this evidence, it is conceivable that this mixture can be used in addition to the GFD mostly in cases of contamination, as its effectiveness limit (2 g) is very low.

Murray et al. (2022) conducted another phase II, randomized, double-blind, placebo-controlled study to assess the efficacy and safety of a 1,200 mg dose of latiglutenase (herein named as IMGX003) in 43 celiac patients exposed to 2 g of gluten per day for six consecutive weeks. The authors observed reductions in both intestinal mucosal damage and symptoms. Furthermore, measurement of gluten immunogenic peptides in the urine indicated 95% degradation of gluten in the stomach by latiglutenase. Another phase II study (NCT04243551) conducted over a period of 6 months evaluated the efficacy, safety, and tolerability of latiglutenase in patients who adhered to the GFD but remained symptomatic; this trial was terminated but the results are not yet published. Entero Therapeutics, the biopharmaceutical company leading the development of latiglutenase, announced on its website that the U.S. Food and Drug Administration (FDA) had reviewed the development plan for this molecule and that phase III studies were expected to begin in early 2025; if the expected endpoints can be achieved, this new pharmacological strategy is expected to be available in the market by 2027.

Another molecule under development is TAK-062, which was previously known as Kuma062 before Takeda Pharmaceutical Company acquired PvP Biologics and its studies. TAK-062 is a highly potent endopeptidase designed *in silico*, derived from the bacterial enzyme kumamolisin-As from *Alicyclobacillus sendaiensis*, and obtained from its precursor Kuma030. *In vitro*, it degraded more than 99% of 3 g and 9 g of gluten within 10 min. The phase I study showed that oral administration of 100 mg, 300 mg, and 900 mg in both healthy and celiac subjects was well tolerated and resulted in an average degradation between 97% and more than 99% within 20–65 min after consuming complex meals containing 1–6 g of gluten. Additionally, the effect of a 7-d pretreatment with 20 mg of esomeprazole was evaluated but no significant differences emerged, indicating that TAK-062 could be safely used in combination with proton pump inhibitors, which are often taken by celiac patients (Pultz et al., 2021). Currently, a phase II, randomized, double-blind, placebo-controlled study is underway to evaluate the efficacy and safety of varying doses of this molecule in celiac patients over the age of 12 who are attempting to follow the GFD; the study was completed by mid-2025 (NCT05353985 in ClinicalTrials.gov).

Although enzymatic therapies represent one of the most advanced pharmacological strategies, the clinical results have been inconsistent, with the results of completed and published clinical studies not supporting an improvement in clinical remission. The efficacies of these therapies may be limited by the amounts of ingested gluten, gastric conditions, and incomplete degradation of peptides. Moreover, none of the compounds have demonstrated sufficient efficacy to allow unrestricted gluten consumption, and no enzyme-based therapy has received regulatory approval for CD. However, these enzymes may improve the responses of patients to low gluten exposure, such as unintentional ingestion due to contamination, thus showing potential as supplements to the GFD (Kivelä et al., 2021).

### 6.3 Blockage of intestinal permeability

The overregulation of zonulin causes the apical protein complexes between enterocytes, called tight junctions, to lose their integrity and result in increased intestinal permeability. Currently, the only molecule capable of acting at this level as a zonulin antagonist is larazotide acetate, also known as AT-1001 or INN-202. It is a synthetic peptide consisting of eight amino acids (Gly-Gly-Val-Leu-Val-Gln-Pro-Gly) with local action that has been shown to reduce intestinal permeability by improving tight junction assembly and actin rearrangement both *in vitro* and *in vivo* (Gopalakrishnan et al., 2012). Its safety and effects have been evaluated in both phase I and phase II studies, and it is the only drug candidate to have also reached phase III studies. Upon oral administration, it was evaluated in a randomized, double-blind phase I study: for 3 d, 14 celiac patients received 12 mg of larazotide, while seven patients received the placebo. Each patient was then given 2.5 g of gluten for 1 d, and the percentage of patients who exhibited GI symptoms was lower in the larazotide group compared to the control group. No severe adverse effects were reported. However, variation in intestinal permeability, measured through the lactulose/mannitol urinary ratio (LAMA), did not differ significantly between the two groups (Paterson et al., 2007). Subsequently, 86 celiac patients on a GFD were recruited for the phase IIa study: the LAMA ratios did not differ between the treatment groups, but the severity of gluten-induced GI symptoms was reduced even with lower doses of larazotide (Leffler et al., 2012). Similar results were obtained from a multicenter, double-blind phase IIb study involving 184 patients: for the same LAMA ratios between treatment groups, only 1 mg of larazotide administered three times a day (and not higher doses) was found to prevent symptoms induced by 2.7 g of gluten over 24 h. For similar low doses of larazotide, a statistically significant reduction in the anti-tTG2 antibody level was also observed (Kelly et al., 2013).

Leffler et al. (2015) conducted a multicenter, randomized, double-blind, 12-week study involving 342 patients with persistent CD symptoms despite a strict GFD and found that the lowest dose of larazotide (0.5 mg administered three times a day) significantly reduced GI disturbance, while doses of 1 mg or 2 mg did not differ from the placebo. The exact mechanism of action of this protease remains unclear. The currently most accepted hypothesis is that larazotide is easily digested into smaller peptide fragments, some of which may have partial antagonistic activities against zonulin. In 2019, phase III of the CedLara® (celiac disease larazotide) trial commenced with the aim of evaluating the effectiveness and safety of larazotide acetate for relieving persistent symptoms in celiac patients despite a GFD. This trial was registered under the number NCT03569007 in ClinicalTrials.gov (status: terminated) and was a randomized, double-blind, placebo-controlled study. The first part of the study consisted of a 5-week screening period to establish patient eligibility; subsequently, the second phase of the double-blind treatment lasted over a total of 24 weeks. The study included 525 patients who were equally divided into three dosage groups: larazotide 0.25 mg, larazotide 0.5 mg, and placebo. However, 9 Meters Biopharma, Inc., the main sponsor of CedLara®, announced suspension of the study on 21 June 2022 as it was determined that the additional number of patients needed to achieve a significant clinical outcome between placebo and larazotide would be too high and that the costs would be unsustainable. Despite the favorable safety profile and some improvement in GI symptoms, larazotide has shown inconsistent efficacy across clinical trials. The termination of the phase III CedLara® study further illustrates the challenges of translating early clinical signals into meaningful therapeutic benefits. At present, larazotide cannot be considered as an established treatment for CD.

### 6.4 TG2 inhibition

TG2 is a ubiquitously expressed protein with many functions, including the ability to catalyze post-translational modifications through the incorporation and deamidation of glutamine (Sulic et al., 2015). It is therefore a key enzyme in the pathogenesis of CD, as the deamidation of gluten peptides leads to the formation of a thioester intermediate between a cysteine residue in the active site of the TG2 catalytic core and a glutamine residue on the peptide’s side chain, which is then converted to glutamic acid. Some preclinical studies revealed that inhibition of TG2 leads to positive effects by blocking the gliadin-induced proliferation of gliadin-specific T cells, preventing the increase in activated T cells (Molberg et al., 2001), and modulating the intestinal epithelial permeability functions *in vitro* (Rauhavirta et al., 2011). In fact, in a transgenic HLA-DQ8 mouse model overexpressing *IL-15* in the intestinal epithelium and *lamina propria mucosae*, two different pharmacological inhibitors of TG2 (named ERW1041E and CK805) prevented the development of villous atrophy induced by gluten (Abadie et al., 2020).

To date, ZED1227 is the only highly specific and irreversible TG2 inhibitor that has been demonstrated to be safe, effective, and beneficial against intestinal inflammation upon oral administration. In particular, Schuppan et al. (2021) conducted a phase II study to test the efficacies of three doses of ZED1227 for preventing gluten-induced mucosal changes in individuals with well-controlled CD. The trial involved 163 patients between the ages of 18 and 65 years, who were divided into four fairly homogeneous groups of 41 subjects to receive either 10 mg, 50 mg, or 100 mg of ZED1227, or a placebo. The trial, which concluded in February 2020, lasted 6 weeks and showed that ZED1227 reduced intestinal mucosal damage in the patients, especially in those who received 50 mg and 100 mg, without reports of serious adverse reactions.

Dotsenko et al. (2024) reported the efficacy of ZED1227 based on molecular histomorphometric evaluations, including remodeling analysis, along with transcriptomic analysis of the intestinal mucosa. They performed two duodenal biopsies in 58 celiac individuals adhering to a GFD: one before and one after a 6-week gluten provocation test combined with a daily treatment of 100 mg ZED1227. The primary objective was to assess the effectiveness of ZED1227 in preventing gluten-induced mucosal damage at the transcriptomic level; the results showed that the tested dose effectively inhibited nearly all gluten-induced changes in gene expression to effectively and specifically prevent gluten-induced mucosal damage. The authors emphasize that HLA-DQ2.5 genetics should also be taken into account and that it may be necessary to adjust the dose or dosing interval of ZED1227 to achieve optimal efficacy. However, further studies are required to confirm this hypothesis. Finally, gene expression changes associated with CD were observed despite a strict GFD, indicating that complete avoidance of gluten is almost impossible; therefore, therapy based on TG2 inhibition may be considered in combination with a GFD. Another inhibitory strategy targeting TG2 activity may act through a redox mechanism involving the formation of an allosteric disulfide bond between two cysteine residues (Cys370 and Cys371) of this enzyme (Stamnaes et al., 2010). Intriguingly, disulfiram, a drug approved by the FDA for alcohol abuse, was found to inhibit TG2 activity *in vitro*, probably through this mechanism (Palanski and Khosla, 2018). Although ZED1227 represents one of the most promising therapeutic candidates, the available evidence is limited to relatively short-term gluten-challenge studies. The long-term safety, consequences of chronic TG2 inhibition, and its efficacy under real-life dietary conditions remain to be established. Therefore, further phase III studies are required before clinical implementation can be considered.

### 6.5 Cathepsin inhibitors

Cathepsins are enzymes classified into several families (serine proteases, cysteine proteases, and aspartic proteases) and are divided into 15 classes. They are activated in the acidic environment of lysosomes and, like other enzymes, are essential for the normal physiological functions of the body, such as digestion, blood coagulation, innate immunity, complement activation, apoptosis, and autophagy. Dysregulated cathepsins have been implicated in the onset of various autoimmune and inflammatory diseases (Patel et al., 2018). Based on this evidence, cathepsin inhibitors have been proposed as a potential strategy for treating various pathologies, including CD, for which cathepsin S inhibitors have been primarily considered. Cathepsin S is specifically expressed by the APCs, where it mediates proteolysis of the invariant chain, an intracellular protein that prevents intracellular loading of the autoantigen during the early stages of development of the MHC class II (MHC-II) molecules in APCs. Later, when the MHC-II molecules reach maturity at the intracellular level, cathepsin S cleaves the invariant chain to allow antigen loading. The activity of this enzyme is crucial for the normal function of MHC-II in presenting the processed antigens to CD4^+^ cells.

Petesicatib (also known as RO5459072 and RG7625) is a cathepsin S inhibitor developed to interfere with this mechanism. The first clinical trial on this drug candidate demonstrated a reduction in the maturation of B cells and DCs presenting MHC-II (Theron et al., 2017). The effects of petesicatib (100 mg b.i.d. or 200 mg daily) on the immune responses to a 13-d gluten challenge were tested in a randomized, double-blind, placebo-controlled, multiple dose, parallel study involving 19 participants with CD (NCT02679014, status: completed). The pharmacodynamic findings suggested that petesicatib had some beneficial effects, such as decreased intestinal permeability and decreases in the numbers of circulating B cells, CD4^+^ T cells, and CD8^+^ T cells compared to baseline. Nevertheless, the absence of clear effects on the responses to the gluten challenge in both arms of the study led the researchers to conclude that inhibition of cathepsin S may not be an effective treatment strategy for CD (Bentley et al., 2025). Despite the strong biological rationale, cathepsin S inhibition remains an early-stage therapeutic approach. The negative results observed during the gluten challenge studies suggest that modulation of antigen processing alone may be insufficient to prevent disease activation.

### 6.6 HLA-DQ2/DQ8 blocking

To prevent the immunotoxic cascade of adaptive immunity in CD, HLA-DQ2/DQ8 blockers have been considered. These can prevent the interactions of the APCs, which process and present the immunogenic gluten epitopes, with the TCRs of the CD4^+^ helper T cells. Cyclic and dimeric peptides have been designed to compete with gliadins and block the HLA-DQ2 binding site. Cyclic peptides have epitopes similar to gliadins, and their cyclization is achieved through disulfide bonds or polyethylene glycol. The effects of cyclization and spacer length in cyclic peptides on DQ2 binding and T cell recognition were analyzed, which showed inhibition of peptide-DQ2 recognition by the TCRs. Similarly, dimeric peptides obtained by dimerizing the peptide sequence with lysine side chains have also shown effective blocking (Xia et al., 2007). However, one aspect that raises many doubts about the possible use of this strategy is the ubiquity of the HLA system and its role in various immune responses, along with the potential interference by peptides in this function. Therefore, the implications of these results for CD treatment are highly debatable (Yoosuf and Makharia, 2019).

By immunizing rabbits with multiepitope gluten pHLA-DQ2.5 and performing multidimensional optimization, a neutralizing antibody similar to the TCR was generated that broadly recognizes over 25 gluten-pHLA-DQ2.5 complexes. This antibody was named DONQ52, and structural analyses show that the proline- and glutamine-rich sites of the gluten epitopes are flexibly recognized by multiple tyrosine residues present in the antibody, leading to broad reactivity. DONQ52 was tested *in vitro* and in HLA-DQ2.5 transgenic mice, which showed favorable pharmacokinetics with high bioavailability after subcutaneous administration compared to other synthesized antibodies; it was also found to block gluten immunity without affecting systemic immunity (Okura et al., 2023).

Based on these results, an *in vivo* study was subsequently approved and completed to evaluate the ability of DONQ52 to inhibit T cell responses to the most immunogenic gluten peptides in celiac patients. Hardy et al. (2024) enrolled 44 HLA-DQ2.5+ celiac patients, who consumed foods containing wheat, barley, or rye for 3 d and underwent blood sampling before and 6 d after the test. Then, mononuclear cells were isolated from the peripheral blood and tested in an immunoassay to assess responses to gluten peptides, including a series of immunodominant T cell epitopes. DONQ52 (4 μg/mL and 40 μg/mL) was more effective in reducing T cell responses to various gluten peptides compared to pan-HLA-DQ antibody blockade. At the same time, the antibody had no effect on T cell responses to antigens other than gluten. Thus, this antibody can significantly block the HLA-DQ2.5-restricted T cell responses to the most immunogenic gluten peptides; a multicenter, randomized, double-blind, and placebo-controlled phase I study to evaluate the safety, tolerability, and pharmacokinetics of DONQ52 in well-controlled CD patients (registered in ClinicalTrials.gov with the number NCT05425446, status: completed) was approved in 2022 but the results are not yet available. Although DONQ52 represents an innovative and highly specific approach, its clinical efficacy and long-term safety have not yet been demonstrated. The absence of published phase I results, together with the complexity and cost of antibody-based therapies, currently limit its translational applicability. Therefore, HLA-DQ blockade should still be considered an experimental therapeutic strategy.

#### 6.7 Gluten tolerance

Among the most studied pharmacological approaches are those aimed at restoring immune tolerance to gluten in people with CD, as these directly target the underlying cause of the disease and could potentially allow safe reintroduction of gluten into the diet. Unlike in many other autoimmune diseases, considerable progress has been made in CD because the antigens that activate pathogenic T cells have been characterized in detail. Furthermore, gluten-specific T cells are a stable population that can maintain specificity and persist for decades in the blood and intestines of patients with the disease (Risnes et al., 2018). Given this information, potential tolerogenic therapies that can induce immune tolerance in cases of pathological or unwanted activation of normal immune responses, such as the use of vaccines to desensitize adaptive immunity, are being studied (Horton et al., 2017).

The most studied tolerogenic therapy for CD is a therapeutic vaccine called Nexvax2 developed by the company ImmuSanT. It is composed of three patented immunodominant gliadin peptides, namely, NPL001, NPL002, and NPL003, each consisting of 15–16 amino acids. The target of this vaccine is the HLA–DQ2.5–epitope–TCR complex that connects the APCs to the gluten-reactive CD4^+^ T cells. In two phase I studies with 82 (Goel et al., 2017) and 36 (Daveson et al., 2017) celiac patients separately, intradermal administration of the vaccine initially caused symptoms, including diarrhea and nausea, similar to those triggered by gluten ingestion. However, with subsequent doses or gradual dose escalation, the symptoms did not differ from those experienced by participants who received the placebo. Furthermore, it was noted that Nexvax2 reduced T cell responses and that the duodenal histology remained stable or tended to improve even after oral administration of 9 g of gluten per day. An increase in serum cytokine levels, particularly IL-2, was detected 2–6 h after the injection (Goel et al., 2019). After establishing the positive clinical and immune system-modifying effects following vaccination, a multicenter phase II study called RESET CeD was initiated in June 2018; the primary aim of this study was to evaluate the efficacy of the vaccine in protecting against gluten-induced symptoms following a crossover, double-blind bolus food challenge with 6 g of gluten or placebo (NCT03644069 in ClinicalTrials.gov; status: unknown; the study is past its completion date but the status has not been verified in more than 2 years). However, the study was discontinued by the manufacturer one year after commencement as an interim analysis showed that the vaccine did not provide statistically significant protection from gluten-induced symptoms (Tye-Din et al., 2023). This finding suggests that suppressing the immune responses induced by Nexvax2 at the administered dose was insufficient to significantly modify the digestive symptoms after reintroducing gluten to the diet.

Among the other antigen-specific approaches under development, one involves loading disease-relevant peptide-MHC complexes directly onto synthetic nanoparticles to suppress the T cells and regulatory B cells (Clemente-Casares et al., 2016). Another strategy uses red blood cells coupled with gluten by considering that when circulating erythrocytes undergo apoptosis (a naturally tolerogenic process), the immune system recognizes the attached antigen and triggers a tolerogenic response (Grimm et al., 2015). This technology was exploited to create KAN-101, a liver-targeting glycosylation signature conjugated to a deaminated gliadin peptide designed to induce immune tolerance to gliadins (Murray et al., 2023). This drug candidate was tested for safety and tolerability in a phase I clinical trial involving 41 HLA-DQ2.5-genotype celiac patients on a GFD for at least 12 months at the start of the study. The trial was divided into two parts: part A was an open-label single ascending dose study of intravenous KAN-101 (from 0.15 mg/kg up to 1·5 mg/kg), followed by a single-blind part B where three escalating doses (0.15 mg/kg, 0.3 mg/kg, and 0.6 mg/kg) of the drug candidate or placebo were administered, followed by a 3-d oral gluten challenge (9 g per day) 1 week after dosing completion. The study concluded with the finding that KAN-101 has an acceptable safety profile in celiac patients, with no dose-limiting toxicity and no maximum tolerated dose. Additionally, rapid systemic clearance of the drug was noted, with no accumulation after repeated doses (Murray et al., 2023).

A phase IIa, multicenter, double-blind, placebo-controlled study (SynCeD study; NCT06001177; status: completed) aiming to evaluate whether KAN-101 protects against gluten-induced duodenal histological changes and to confirm its safety and tolerability in celiac patients following a GFD was completed in January 2025; the results of this study are yet to be published. Antigen-specific tolerance remains an attractive therapeutic concept because it directly targets the pathogenic immune response. However, discontinuation of Nexvax2 despite encouraging results in early studies highlights the complexity of restoring immune tolerance in CD. Newer approaches like KAN-101 are in the early stages of clinical development, and their long-term efficacy and durability are still unknown.

### 6.8 Anti-IL-15 therapy

IL-15 and its downstream signaling pathway represent interesting pharmacological targets: they are directly involved in the cellular processes underlying the pathophysiology of CD as they are upregulated in both the intestinal epithelial cells and *lamina propria mucosae* (Di Sabatino and Corazza, 2009). A fully human monoclonal antibody called AMG 714 has been developed, which binds to and inhibits all forms of IL-15. It was tested in two randomized, double-blind, placebo-controlled phase IIa studies. The first study included 64 celiac patients on a GFD; however, only 49 patients were able to receive 150 mg or 300 mg of AMG 714 or the placebo subcutaneously every 2 weeks for a total of six doses. The primary outcome was not achieved as the monoclonal antibody did not prevent damage to the small intestine mucosa during a gluten challenge of 2–4 g per day. However, despite increases in the numbers of IELs in all groups, the increase was less pronounced in those who received 300 mg of AMG 714. Additionally, among the typical symptoms of the disease, diarrhea improved with both doses of the drug, and no serious adverse effects were reported (Lähdeaho et al., 2019).

The second study involved 28 patients with RCD II as it was hypothesized that IL-15 overexpression in the intestine in this specific case contributes to expansion of the aberrant IEL subpopulation characteristic of this disease (Hujoel and Murray, 2020). During the study, 19 patients received 8 mg/kg of AMG 714 and nine patients received a placebo intravenously over 10 weeks for a total of seven doses. The primary outcome, which was the reduction of aberrant IELs, was not achieved. However, a statistically significant difference was observed in the percentage of patients who experienced at least one episode of diarrhea per week during the study, with a decreasing percentage in the AMG 714 group and an increasing percentage in the placebo group. Serious adverse events were recorded, including tuberculosis, pneumococcal infection, and cerebellar syndrome, in 26% of patients in the AMG 714 group and 11% of patients in the placebo group (Cellier et al., 2019). Another phase IIb, randomized, double-blind, placebo-controlled study (NCT04424927; status: completed) to evaluate the safety and effects of AMG 714 (referred to as PRV-015 herein) on symptoms among patients with NRCD was started in 2020 and ended in 2024; the study aimed to explore this pharmacological strategy as a support for the GFD, but the results are not yet published.

In addition to direct inhibition, the signaling pathway of IL-15 has been considered as a potential pharmacological target. The IL-15 pathway involves three different receptor chains: the γ chain of the common cytokine receptor that is shared by five other γ-chain cytokines, including IL-21; the IL-2/IL-15 receptor β (IL-15Rβ) shared with IL-2; a third subunit of the IL-15 receptor, i.e., IL-15Rα, which is specific to IL-15 (Waldmann, 2013). BNZ-2 is a peptide that binds to the common γ chain and has been shown to specifically inhibit IL-15- and IL-21-mediated activation of human intraepithelial cytotoxic T cells. It represents a potential future drug candidate for CD therapy since a higher percentage of patients overexpress both IL-15 and IL-21 (57.1%) rather than one of the two cytokines selectively (14.3%) (Ciszewski et al., 2020). The humanized monoclonal antibody Mik-β-1 targets the IL-2/IL-15Rβ cytokine receptor subunit; its efficacy was evaluated in a phase I study involving five patients with RCD who received one dose of the drug every 3 weeks. The study, registered under number NCT01893775 in ClinicalTrials.gov, was completed in 2019 but the results are currently unavailable.

For the treatment of RCD, tofacitinib, a JAK1 and JAK3 inhibitor has shown beneficial effects in transgenic mice mimicking the pathological condition. It is currently used in clinical settings as a therapy for rheumatoid arthritis, psoriatic arthritis, and ulcerative colitis (Yokoyama et al., 2013). Grewal et al. (2022) used this drug off-label in a middle-aged patient who developed RCD II postpartum; they administered 10 mg of the drug twice daily, and a follow-up gastroduodenoscopy was performed after 4 months of the trial, which demonstrated an improvement in the lesions. The dose was then reduced to 5 mg twice daily to minimize the risk of adverse effects, which were not observed in this case report. Although IL-15 represents a biologically attractive target, clinical studies have thus far yielded modest or negative results, particularly regarding mucosal healing. In addition, the occurrence of serious adverse events and the potential risks associated with systemic immune modulation may limit the applicability of these therapies outside selected patient populations, such as those with RCD.

### 6.9 Nanoparticles

Another possible therapeutic approach for CD is the use of nanoparticles. Freitag et al. (2020) analyzed the safety and efficacy of a negatively charged poly-lactic-co-glycolic acid nanoparticle with encapsulated gliadin (TIMP-GLIA) in modulating the immune responses in three transgenic mouse models. The injection of TIMP-GLIA suppressed the inflammatory responses of the T cells and B cells, along with reducing gluten-dependent enteropathy. Based on the experimental data, it is believed that TIMP-GLIA is absorbed by the APCs in the spleen and liver, which process and cross-present a series of gliadin peptides in a tolerogenic manner (Kelly et al., 2021).

A completed phase I study (NCT03486990 in ClinicalTrials.gov) reported the safety, tolerability, and pharmacokinetic evaluations of TIMP-GLIA in 23 individuals. According to the results, the drug candidate was found to be safe, although some participants experienced mild adverse events. Based on this finding, a phase II, double-blind, randomized, proof-of-concept trial (NCT03738475 in ClinicalTrials.gov) involving 34 patients affected by the disease was completed in August 2020. The participants received two intravenous infusions of 8 mg/kg (up to a maximum of 650 mg) of TIMP-GLIA or a placebo; the following week, they began to consume wheat gluten for 14 d. Patients treated with TIMP-GLIA showed a gluten-specific T cell immune response after a gluten test of 6–12 g per day, which was six times lower than the responses of the patients in the placebo group (Kelly et al., 2021).

The ClinicalTrials.gov website also lists another phase II, randomized, double-blind, placebo-controlled study sponsored by Takeda (identification number NCT04530123; status: completed) that aimed to evaluate the efficacy and safety of TAK-101, the name designated by the sponsor to TIMP-GLIA, for preventing gluten-specific T cell activation in patients with CD who follow a GFD. Nanoparticle-based therapies represent an innovative strategy for inducing antigen-specific tolerance, but clinical experience remains extremely limited. Current evidence has been derived mainly from animal models and early-phase studies, and important questions regarding the long-term efficacy, manufacturing complexity, costs, and large-scale implementation remain unanswered.

## 7 New therapeutic hypothesis and future directions

Beyond dietary management, there is increasing attention on pharmacological strategies capable of targeting the key molecular nodes underlying CD pathogenesis, particularly oxidative stress, epithelial barrier dysfunction, and dysregulated immune responses. In this context, drug repurposing represents a particularly attractive approach as it allows the identification of novel therapeutic indications for approved compounds, thereby reducing both development time and associated costs while relying on well-established safety profiles (Manai et al., 2023b; Saidu et al., 2019). Among these candidates, dimethyl fumarate (DMF) has emerged as a molecule of considerable interest.

DMF is currently approved by both the European Medicines Agency and FDA for the treatment of psoriasis and relapsing–remitting multiple sclerosis (RRMS), with its first clinical formulation (Fumaderm®) being introduced in the 1990s and a more recent formulation (Tecfidera®) being approved in 2013 (Saidu et al., 2019). Following oral administration, DMF is rapidly and extensively metabolized in the GI tract into monomethyl fumarate (MMF), which represents the primary bioactive metabolite detectable in systemic circulation and reaches a peak concentration within 2–3 h (Litjens et al., 2004; Ghoreschi et al., 2011). This rapid presystemic metabolism involving enzymatic reactions, such as hydrolysis and oxidation, also leads to the production of methanol, a compound associated with local GI irritation. Both DMF and MMF can additionally form glutathione (GSH) adducts (GS-DMS and GS-MMS) within the intestinal mucosa, reflecting a rapid intracellular redox interaction that is central to their mechanisms of action (Manai et al., 2023b).

At the molecular level, DMF exerts pleiotropic effects that converge on the modulation of cellular redox homeostasis and inflammatory signaling. A key event here is the disruption of the Keap1–Nrf2 complex that allows nuclear translocation of Nrf2 and subsequent transcriptional activation of the antioxidant response element (ARE)-driven genes, including *TP53*, *NQO1*, *GCLC*, and *HO-1* (Canning et al., 2015). This pathway is increasingly recognized as a central regulator of not only oxidative stress responses but also autophagy and epithelial barrier integrity—processes that are profoundly altered in CD. In parallel, DMF interferes with additional proinflammatory pathways, including inhibition of caspase-1 and hypoxia-inducible factor-1α (Liu et al., 2016), and indirect suppression of NF-κB signaling through HO-1 and sirtuin-1-mediated mechanisms (Gillard et al., 2015). Collectively, these effects result in a coordinated downregulation of proinflammatory cytokine production and a shift toward a more tolerogenic immune environment.

Importantly, the relevance of these mechanisms to CD is supported by the central roles of oxidative stress and impaired autophagy in gliadin-induced toxicity. In particular, peptides such as p31-43 have been shown to induce reactive oxygen species (ROS) accumulation, disrupt vesicular trafficking, and alter autophagic flux, ultimately contributing to epithelial damage and inflammation. In this context, pharmacological activation of the Nrf2/Keap1/ARE axis may represent a strategic point of intervention capable of restoring the redox balance and limiting the downstream inflammatory cascades (Bresciani et al., 2023). Consistent with this hypothesis, several lines of evidence highlight the protective role of Nrf2 signaling in intestinal inflammation. Experimental models of dextran-sulfate-sodium-induced colitis have demonstrated that Nrf2 deficiency increases susceptibility to epithelial damage and inflammatory responses (Khor et al., 2006), while its activation contributes to the maintenance of intestinal barrier homeostasis (Piotrowska et al., 2021). In CD-specific contexts, Nrf2 activation has been associated with beneficial effects through different approaches: wheat-germ-derived peptides, resveratrol, conjugated linolenic acid, and 5-aminosalicylic acid have all been shown to reduce oxidative stress and epithelial injury via modulation of this pathway (Wang et al., 2022; Yu et al., 2022; Bergamo et al., 2016; Kang et al., 2017). These findings collectively support the rationale for targeting Nrf2 as a convergent mechanism in gluten-related disorders.

Beyond its intracellular effects, DMF may also influence disease-relevant processes through modulation of the gut microbiota, an emerging factor in CD pathogenesis. Initial observations by Eppinga et al. (2017) reported an increase in *Saccharomyces cerevisiae* levels in fecal samples from psoriasis patients treated with DMF, alongside evidence of direct growth promotion *in vitro*. Additional studies in RRMS cohorts have described broader compositional shifts, including reductions in *Clostridiales*, *Lachnospiraceae*, *Veillonellaceae*, *Firmicutes*, and *Fusobacteria*, with a concomitant increase in *Bacteroidetes* (Katz Sand et al., 2018) and persistent decreases in *Clostridium* species even after treatment discontinuation (Ferri et al., 2023). However, these findings are not entirely consistent as other studies have reported minimal or only transient microbiota alterations following DMF administration (Storm-Larsen et al., 2019). Notably, changes in the microbial compositions—particularly overgrowth of otherwise commensal organisms such as *S. cerevisiae*—may also contribute to the GI adverse effects commonly observed during DMF therapy, including nausea, abdominal pain, and diarrhea (Herbrecht and Nivoix, 2005).

Although these side effects are generally manageable and reversible through dose adjustments or modified administration schedules (Kofler et al., 2019), they represent a relevant limitation for long-term use in chronic conditions such as CD. In this regard, alternative formulations such as diroximel fumarate have been developed to improve GI tolerability while maintaining comparable efficacy (Yadav et al., 2026). Similarly, direct administration of MMF has been proposed as a strategy to reduce local intestinal toxicity, although some evidence suggests that the parent compound DMF may retain superior biological activity in certain contexts (Piotrowska et al., 2021).

From an immunological perspective, DMF may further contribute to disease modulation by promoting a tolerogenic phenotype in APCs and by inhibiting T cell activation and differentiation (Djedovic et al., 2019; Mazzola et al., 2019). These effects are particularly relevant in CD, where aberrant activation of both innate and adaptive immune responses drives tissue damage. The ability of DMF to simultaneously target oxidative stress, immune activation, and epithelial dysfunction positions it as a candidate capable of acting on multiple interconnected pathogenic pathways rather than a single molecular target. Nevertheless, despite this strong mechanistic rationale, clinical translation remains limited, and evidence in CD-specific settings is still lacking. Previous attempts to repurpose other anti-inflammatory agents commonly used in inflammatory bowel diseases, such as budesonide and mesalazine, have yielded mixed results. A randomized, double-blind, phase II trial demonstrated that budesonide (9 mg/d) did not improve villous atrophy compared to placebo in CD patients after both 8 and 52 weeks of treatment (Newnham et al., 2021). Conversely, mesalazine has shown some capacity to reduce oxidative stress and modulate PPAR-γ expression in *ex vivo* duodenal biopsy cultures from CD patients (Benedetti et al., 2018), although its clinical relevance remains uncertain.

Together, these observations highlight both the potential and limitations of current pharmacological strategies beyond the GFD. While DMF and other Nrf2-activating compounds offer a promising avenue by targeting central disease mechanisms, future research efforts should move toward a more integrated understanding of CD as a network disorder involving redox imbalance, immune dysregulation, microbiota alterations, and epithelial barrier dysfunction. In this framework, therapeutic approaches capable of simultaneously modulating these interconnected processes—rather than acting on single pathways—may represent the most effective strategy for advancing CD management in the future.

## 8 Conclusion

CD has long been considered one of the best-characterized immune-mediated disorders, yet this perception is largely misleading. Although the molecular underpinnings of the disease, ranging from the unique biochemical properties of gluten to the central role of TG2 and HLA-DQ-restricted antigen presentation, have been explored extensively, this wealth of knowledge has not been translated into meaningful therapeutic innovations. Continued reliance on the GFD as the sole effective treatment underscores a fundamental disconnect between mechanistic insight and clinical impact. This translational failure reflects a deeper conceptual limitation in the field. Much of the current research paradigm remains reductionist, focusing on isolated components of a highly interconnected system. Targeting single elements, whether these are gluten peptides, TG2 activity, or specific immune pathways, has repeatedly proven insufficient as CD emerges from the convergence of multiple processes, including epithelial barrier dysfunction, innate immune activation, adaptive immune responses, and environmental modifiers such as the gut microbiota. As a result, many promising strategies have shown efficacy in controlled experimental settings but have failed to achieve robustness, safety, or reproducibility in clinical contexts. Moreover, it is likely that the extent of inter-individual heterogeneity has been underestimated. The assumption that CD represents a uniform entity has hindered the development of tailored interventions despite clear variability in the genetic background, immune responses, microbiota compositions, and clinical presentations. In this regard, the lack of validated biomarkers capable of stratifying patients or predicting therapeutic responses remains a major obstacle.

A shift in perspective is therefore needed urgently. Future efforts should prioritize integrative, systems-level approaches that move beyond linear cause–effect models and instead capture the dynamic interactions among the host, diet, and microbiota. The integration of multi-omics data, advanced *in vitro* systems such as organoids, and more representative *in vivo* models may provide a more accurate framework for understanding the complexity of CD and identifying actionable targets. At the same time, regulatory barriers, particularly those affecting the development of genetically engineered cereals, must be critically re-evaluated as they currently limit the translational potential of otherwise promising strategies.

Importantly, the notion that the GFD can or should be fully replaced may itself be flawed. A more realistic and potentially more effective paradigm would be the development of adjunctive, combinatorial therapies aimed at reducing gluten immunogenicity, restoring barrier integrity, and selectively modulating immune responses. When tailored to defined patient subsets, such strategies may finally bridge the gap between mechanistic understanding and clinical application. In this context, CD should no longer be viewed as a “solved” condition managed by dietary exclusion but rather as a model system for complex immune–environment interactions that poses unresolved biological and therapeutic challenges. Addressing these challenges will require not only technological advances but also a critical reassessment of the assumptions that have shaped the field to date.
